# SAVI: molecular mechanisms, clinical spectrum and precision medicine approaches beyond type-I IFN

**DOI:** 10.3389/fimmu.2026.1928419

**Published:** 2026-08-31

**Authors:** Michele Manganelli, Paola Cantalice, Jona Papri, Enrico Drago, Nicole De Lucchi, Giulia Accorsi, Chiara Fresia, Simonetta Simonetti, Gabriella Guida, Gerardo Cazzato, Mario Della Mura, Giuseppe Ingravallo, Roberta Caorsi, Stefano Volpi, Francesco La Torre, Marco Gattorno

**Affiliations:** 1 Department of Precision and Regenerative Medicine and Ionian Area, University of Bari Aldo Moro, Bari, Italy; 2 Pediatric Rheumatology Unit, Giovanni XXIII Children’s Hospital, Azienda Ospedaliero Universitaria Consorziale (AOU) Policlinico-Giovanni XXIII, University of Bari Aldo Moro, Bari, Italy; 3 Dipartimento di Neuroscienze, Riabilitazione, Oftalmologia, Genetica e Scienze Materno-Infantili, Università degli Studi di Genova, Genoa, Italy; 4 Clinical and Experimental Immunology, IRCCS Istituto Giannina Gaslini, Genoa, Italy; 5 Gene Therapy Program, Dana Farber/Boston Children’s Cancer and Blood Disorders Center, Harvard Medical School, Boston, MA, United States; 6 Clinical Pathology and Neonatal Screening, Giovanni XXIII Children’s Hospital, Azienda Ospedaliero Universitaria Consorziale (AOU) Policlinico-Giovanni XXIII, University of Bari Aldo Moro, Bari, Italy; 7 Department of Translational Biomedicine and Neuroscience, University of Bari Aldo Moro, Bari, Italy; 8 Section of Molecular Pathology, Department of Precision and Regenerative Medicine and Ionian Area, University of Bari Aldo Moro, Bari, Italy; 9 Rheumatology and Autoinflammatory Diseases Unit, IRCCS Istituto Giannina Gaslini, Genoa, Italy

**Keywords:** anifrolumab, interstitial lung disease (ILD), Janus kinase inhibitors (JAK inhibitors), STING-associated vasculopathy with onset in infancy (SAVI), type I interferonopathy, vasculopathy

## Abstract

Type I interferonopathies (TI-IFN) represent a heterogeneous group of autoinflammatory disorders characterized by upregulated type I interferon (IFN) signaling. Among them, STING-associated vasculopathy with onset in infancy (SAVI) is a rare, severe autoinflammatory disease caused by gain-of-function mutations in the STING1 gene. Mechanistically, these mutations lead to a constitutive activation of the STING protein, resulting in excessive type I interferon production, which drives chronic inflammation and damage to various organs, primarily as cutaneous vasculopathy and progressive interstitial lung disease (ILD). Emerging clinical data indicate that current therapeutic options – including Janus kinase inhibitors (JAKi), biological agents as anifrolumab and solid organ transplantation – for SAVI face limited and often inconsistent long-term efficacy, highlighting a significant unmet need. Consistently, preclinical models highlight that SAVI pathogenesis is not only driven by the interferon storm, but relies also on non-canonical IFN-independent cellular pathways across distinct hematopoietic and non-hematopoietic compartments. This review provides a comprehensive overview of SAVI pathogenesis, from mutational mechanisms and comparative phenotypes to the clinical challenges of advanced interventions. Finally, we discuss future therapeutic prospects and clinical implications, exploring next-generation frontiers such as patient-derived induced pluripotent stem cell (iPSCs) models, hematopoietic stem cell transplantation (allo-HSCT) and gene editing (GE) technologies. The transition from immune - to a mutational-perspective, provides a foundational framework to optimize diagnostic and therapeutic strategies for precision medicine in SAVI patients.

## 1 Introduction

Auto-inflammatory diseases (AIDs) are a group of conditions characterized by an abnormal activation of the innate immune system in the absence of infection due to mutations in genes encoding proteins that play a crucial role in regulating the inflammatory response. Due to their genetic nature, the clinical onset is early, usually within the first decade of life, while cases of adult-onset are rare (1, 2).

The pathogenic potential of interferons (IFNs) was first recognized in the 1970s by Gresser and colleagues (3), when high-dose IFN injections in newborn mice replicated the severe organ damage of viral infections, a process preventable by anti-IFN antibodies (4). More recently, in 2003 Crow and his collaborators (5) linked this mechanism to human disease by demonstrating that Aicardi-Goutières Syndrome (AGS), Systemic Lupus Erythematosus (SLE), and congenital viral infections share a common phenotype driven by dysregulated interferon-alpha (IFNα) production (5).

Most gene mutations implicated in the pathogenesis of interferonopathy affect genes involved in nucleic acid metabolism, recognition, and signal transduction. Indeed, mutations occur in enzymes responsible for preventing the accumulation of DNA, RNA, and hybrid DNA-RNA molecules, including DNA 3’-repair exonuclease 1 (TREX1) and ribonuclease H2 (RNASEH2), which degrade nucleic acids (6, 7), SAMHD1, which limits cytosolic deoxynucleotide (dNTP) availability (8), and adenosine deaminase RNA-specific 1 (ADAR1), which processes endogenous dsRNA, preventing its recognition by the cytosolic receptor IFIH1 (9). In these cases, type I IFN pathway activation is a consequence of a lower detection threshold for cellular nucleic acids sensors. Activating mutations of the nucleic acid receptors IFIH1 and RIG-I, responsible for AGS and Singleton-Merten syndrome, and activating mutations of STING, underlying STING-associated vasculopathy with onset in infancy (SAVI) (10–12), have also been observed. In these syndromes, type I IFN pathway activation results from the constitutive activation of receptors or downstream molecules responsible for signal transduction. Finally, some interferonopathies are caused by mutations in proteins that inhibit the type I IFN response to prevent toxicity from excessive downstream effector molecule activity. Examples include ISG15 and USP18 deficiencies: ISG15 stabilizes USP18, a ubiquitin-specific protease that competitively prevents JAK1 binding to the IFNAR2 subunit of the type I interferon receptor (IFNAR). Mutations in these two proteins lead to uncontrolled activation of the IFN pathway. Recently, a novel mutation responsible for a defect in type I IFN receptor signal regulation has been observed. This mutation (c.1835G>A, p.R612H) affects STAT2, a protein that, when altered, fails to adequately transfer USP18 to IFNAR2, thus preventing USP18 from negatively regulating the type I IFN response, while leaving the type II IFN (IFN- γ) signaling pathway unaffected (13–15).

To bridge these broader mechanisms with specific clinical needs, although the molecular landscape of type I interferonopathies is rapidly expanding and several reports cover general aspects of pathogenesis, clinical features, and current treatments, SAVI remains a significant clinical and therapeutic challenge. This comprehensive narrative review provides a timely overview of SAVI, focusing on the mechanistic impact of STING1 mutations and their associated clinical phenotypes.

To ensure scientific transparency and a comprehensive overview of the field, we conducted a literature search across major electronic databases, including PubMed/MEDLINE, GeneReviews and OMIM, capturing records published up to 2026. Our search strategy accounts a comprehensive Boolean string: (“SAVI” OR “STING-associated vasculopathy with onset in infancy” OR “TMEM173” OR “stimulator of interferon genes” OR “MPYS” OR “type I interferonopathy” OR “type 1 interferonopathies” OR “interferon-related biomarkers” OR “interferon signature” OR “interferon-alpha/beta over-amplification”) AND (“autoinflammatory disease” OR “autoinflammation” OR “innate immunity” OR “interferon-stimulated genes” OR “ISG” OR “cGAS” OR “cyclic GMP-AMP synthase” OR “COPA syndrome” OR “Aicardi-Goutières syndrome” OR “CANDLE” OR “PRAAS” OR “STAT2” OR “USP18” OR “IFIH1” OR “DDX58”) AND (“interstitial lung disease” OR “pulmonary fibrosis” OR “vasculopathy” OR “vasculitis” OR “endothelial toxicity” OR “necroptosis” OR “integrated stress response” OR “UPR” OR “JAK inhibitors” OR “ruxolitinib” OR “baricitinib” OR “tofacitinib” OR “anifrolumab” OR “stem cell transplantation” OR “lung transplantation” OR “gene editing” OR “CRISPR” OR “iPSC”).

Inclusion criteria comprised peer-reviewed original research, clinical guidelines and case reports or series detailing the pathophysiology, biomarkers, and management of SAVI and related type I interferonopathies. Exclusion criteria omitted non-peer-reviewed articles, editorials lacking primary data, conference abstracts without full-text availability, and studies on unrelated conditions. We critically evaluated how current genetic and molecular insights can be leveraged for personalized therapeutic strategies, aiming to optimize the clinical management of patients affected by this rare disease.

## 2 STING1 signaling

### 2.1 Type I interferons: overview and classification

Interferons (IFNs) are a class of inflammatory cytokines involved in the activation and regulation of the immune response, representing the first line of defense against pathogens. The term “interferon” derives from their ability to “interfere” with viral infection. In 1957, Isaacs and Lindenmann observed that the supernatant of cells incubated with influenza virus, when added to another cell culture, could protect the other cells from the pathogen (16). The fact that every nucleated cell can produce IFNs in response to viral infection indicates their crucial role in establishing an “antiviral state” within the cell. Three distinct classes of interferons exist (I-II-III); among these, type I IFNs represent the largest family (16). This family encompasses several proteins, including: IFN-α (comprising 13 molecules), IFN-β, IFN-δ, IFN-ϵ, IFN-κ, IFN-ζ, IFN-τ, and IFN-ω. These proteins are encoded by different genes organized in tandem on chromosome 9p and share a common signaling pathway through a common receptor (17, 18). While the roles of IFNα and IFNβ in the pathogenesis of interferonopathies are well-defined, the contribution of other interferon types is poorly understood (19).

### 2.2 Cytoplasmic DNA sensing and type I interferon signaling

Type I IFN production is induced by the detection of nucleic acids from viral and bacterial pathogens by Pattern Recognition Receptors (PRRs), in the cytoplasm or endosomes of infected cells. PRRs include Toll-like receptors (TLRs), RIG-I-like receptors (RLRs), NOD-like receptors (NLRs), and a growing family of cytoplasmic DNA receptors such as AIM2, cyclic GMP-AMP synthase (cGAS), and γ-IFN-inducible protein 16 (IFI16) 1 (20). The role of these sensors in the pathogenesis of type I interferonopathies (TI-IFN) is now well-established. Specifically, alterations in RLRs (MDA5 and RIG-I) and anomalies in cGAS-mediated DNA recognition pathways have been identified as leading to severe inflammatory clinical presentations. The signal transmitted by RLRs is mediated by the mitochondrial antiviral signaling protein (MAVS), whereas the signal from cGAS and IFI16 is transmitted by the STING protein. Cytoplasmic dsDNA interacts with the cGAS enzyme, which catalyzes the synthesis of cyclic dinucleotide GMP-AMP (cGAMP) (21). cGAMP binds to STING, which, upon activation, leads to profound conformational changes that trigger STING oligomerization, disengagement from anchoring factors (such as STIM1), interaction with trafficking factors (SEC24/23, STEEP) and, finally, incorporation into coatomer protein complex II (COPII) vesicles. Following cGAMP—triggered translocation, Armadillo—like helical domain—containing protein 3 interacts with STING at the Golgi and mediates the recruitment of phosphatidylinositol 4—kinase beta (PI4KB) to synthesize PI4P, which is involved by PI4P—binding proteins in the trafficking of STING from the Golgi to endosomes. Abnormally elevated cellular PI4P has been also shown to induce a cGAS-independent STING activation (22). Other activator of STING are IFI16 and DNA damage response factors ATM and PARP-1, Functionally, *B. abortus*, influenza A virus and streptococcal M protein have been reported to trigger STING independently of cGAS to induce host protection (23–26). STING then translocates from the endoplasmic reticulum (ER) to the ER-Golgi intermediate compartments (ERGIC). At the Golgi, STING undergoes essential post-translational modifications, such as palmitoylation, which are required for the recruitment of downstream signaling molecules (27).

### 2.3 Downstream signaling and biological effector functions

The signal propagates through the phosphorylation of TANK-binding kinase 1 (TBK1) and a family of proteins known as IFN regulatory factors (IRFs), particularly IRF3. In type I dependent-interferon signaling, IRF3 translocates to the nucleus and induces IFNβ transcription, while IRF7 is responsible for IFNα induction and the amplification of the autocrine signaling mediated by type I IFNs (28–30). IRF3 also triggers an IFN-independent pathway that bypasses the need for IFNAR receptor engagement and JAK-STAT signaling. Activated IRF3 homodimers bind directly to IFN-stimulated response element (ISRE) within the promoters of ISG15, IFIT1 and CXCL10 (31, 32). Therefore, excessive activation of the cellular nucleotide receptor system can lead to increased interferon production and, consequently, an inappropriate inflammatory state. Activated STING also triggers the IKK complex, leading to the phosphorylation and nuclear translocation of NF-κB, which directly induces the production of pro-inflammatory cytokines such as TNFα, IL-6 and IL-21 (33).

Basal type I IFN production is essential for maintaining a state of cellular “readiness”. Currently, two distinct functions of the type I IFN pathway are described: antiviral and antiproliferative activity. The effector arm of antiviral activity involves: dendritic cells (DCs), which, upon IFN stimulation, mature, migrate, and enhance antigen presentation; natural killer (NK) cells, which increase their cytotoxic activity and IFN-γ secretion; T lymphocytes, particularly CD4^+^ and CD8^+^ T cells, which increase MHC class I expression, proliferate and differentiate toward the Th1 phenotype; and B lymphocytes, which differentiate into immunoglobulin-secreting cells. Antiproliferative activity is mediated by *c-MYC* downregulation, Crk signaling pathway activation, and the induction of cyclin-dependent kinases (Cdks), leading to G1 phase cell cycle arrest. While antiviral activity is mediated by all IFN types, even at low concentrations and in most cells, antiproliferative activity is highly cell-specific and requires high IFN and receptor expression, as well as adequate binding affinity between them (34–36).

### 2.4 IFNAR receptor complex and JAK-STAT signaling

**Figure 1:**
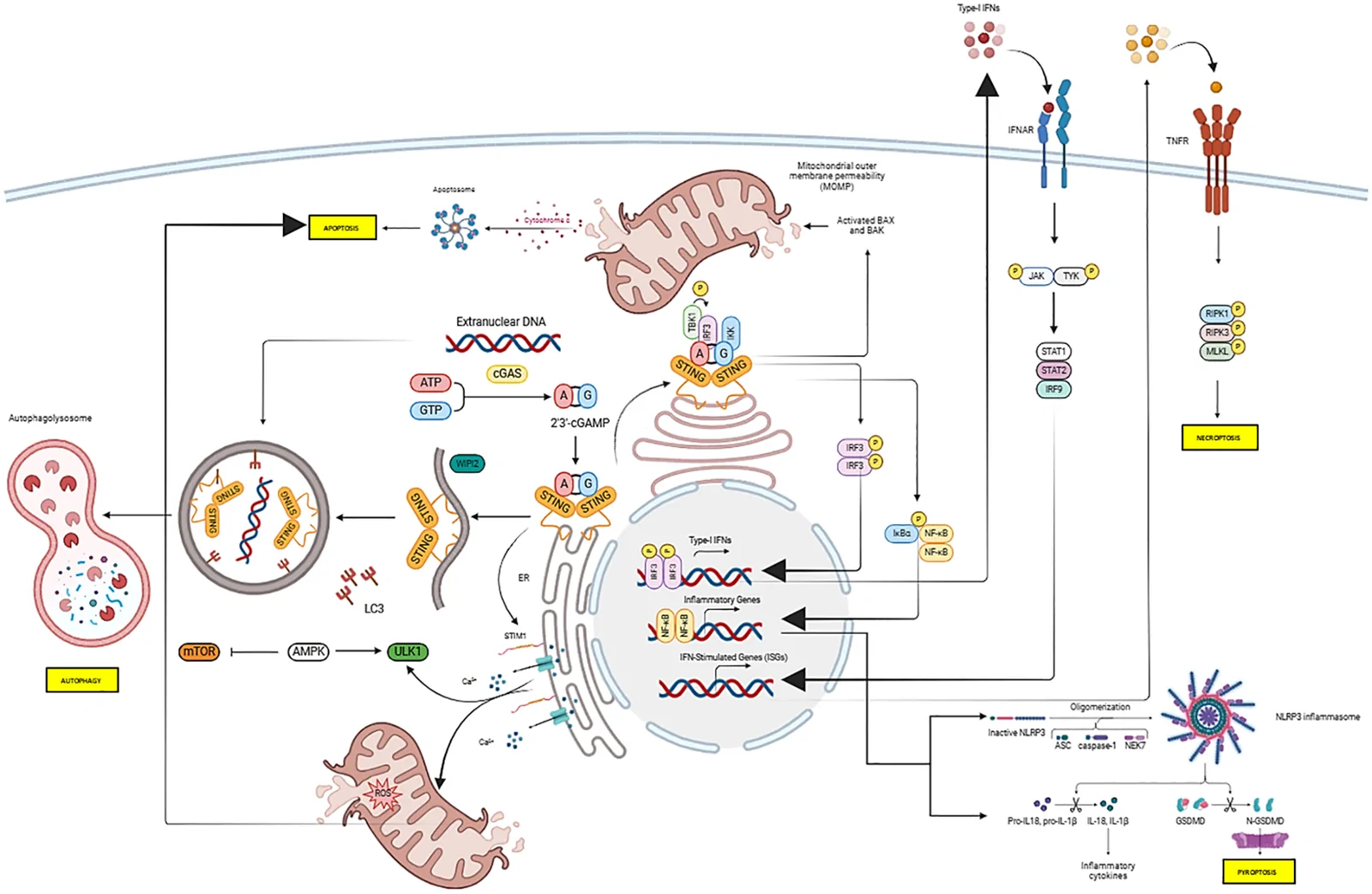
Canonical and non-canonical signaling pathways activated by hyperactive STING in SAVI. Upon binding of 2’3’-cGAMP (synthesized by cGAS from ATP and GTP in response to extranuclear DNA) or via disease-causing mutations, STING translocates from the endoplasmic reticulum (ER) through the Golgi apparatus. In the canonical pathway (center/right), STING recruits TBK1 and IKK, leading to the phosphorylation and nuclear translocation of IRF3 and NF-κB. This drives the transcription of type I interferons and inflammatory genes. Secreted type I interferons bind to the cell-surface receptor (IFNAR), activating the classical JAK-STAT pathway to upregulate Interferon-Stimulated Genes (ISGs). In non-canonical pathways (left and bottom), hyperactive STING engages multiple interferon-independent mechanisms: it modulates the autophagy machinery by the mTOR-AMPK-ULK1 axis, WIPI2, and LC3 to form autophagosomes; it induces mitochondrial outer membrane permeabilization (MOMP) mediated by BAX/BAK, leading to cytochrome c release, apoptosome assembly, and intrinsic apoptosis; it triggers necroptosis through the TNFR-RIPK1/RIPK3/MLKL axis; and it promotes NLRP3 inflammasome assembly (involving ASC, caspase-1, and NEK7), resulting in the maturation of IL-1β/IL-18 and gasdermin D (GSDMD)-mediated pyroptosis. These combined non-canonical pathways drive severe inflammation and T cellopenia independently of the JAK-STAT axis. This figure was created by BioRender.com.

The various type I interferon molecules bind the same heterodimeric receptor, expressed by all nucleated cells and composed of two subunits, IFNAR1 and IFNAR2 (**Figure 1**). Interferon binding to a receptor subunit induces IFNAR1 and IFNAR2 dimerization, phosphorylation of Janus Kinases (JAK1) and TYK2 and activation of STAT family members, forming three main complexes: the pSTAT1 homodimer, the pSTAT3 homodimer, and the pSTAT1/pSTAT2 heterodimer. The latter, together with IRF9, activates the transcription factor IFN-stimulated gene factor 3 (ISGF3), which, by binding to ISRE sequences, induces the transcription of numerous interferon-stimulated antiviral genes (ISGs). The pSTAT1 homodimer regulates IFNγ-activated sequences (GASs) for pro-inflammatory gene transcription (37). The pSTAT3 homodimer, conversely, induces suppression of the type I IFN-mediated response (38).

## 3 STING-associated vasculopathy with onset in infancy

### 3.1 Epidemiology and genetic background

In 2014, STING-associated vasculopathy with onset in infancy (SAVI) syndrome was described (12, 39, 40). SAVI syndrome (#OMIM: 615934) is rare interferonopathy with a prevalence of <1/1.000.000 caused by heterozygous mutations in the *TMEM173* gene, recently renamed *STING1* (5q31.2).

**Figure 2:**
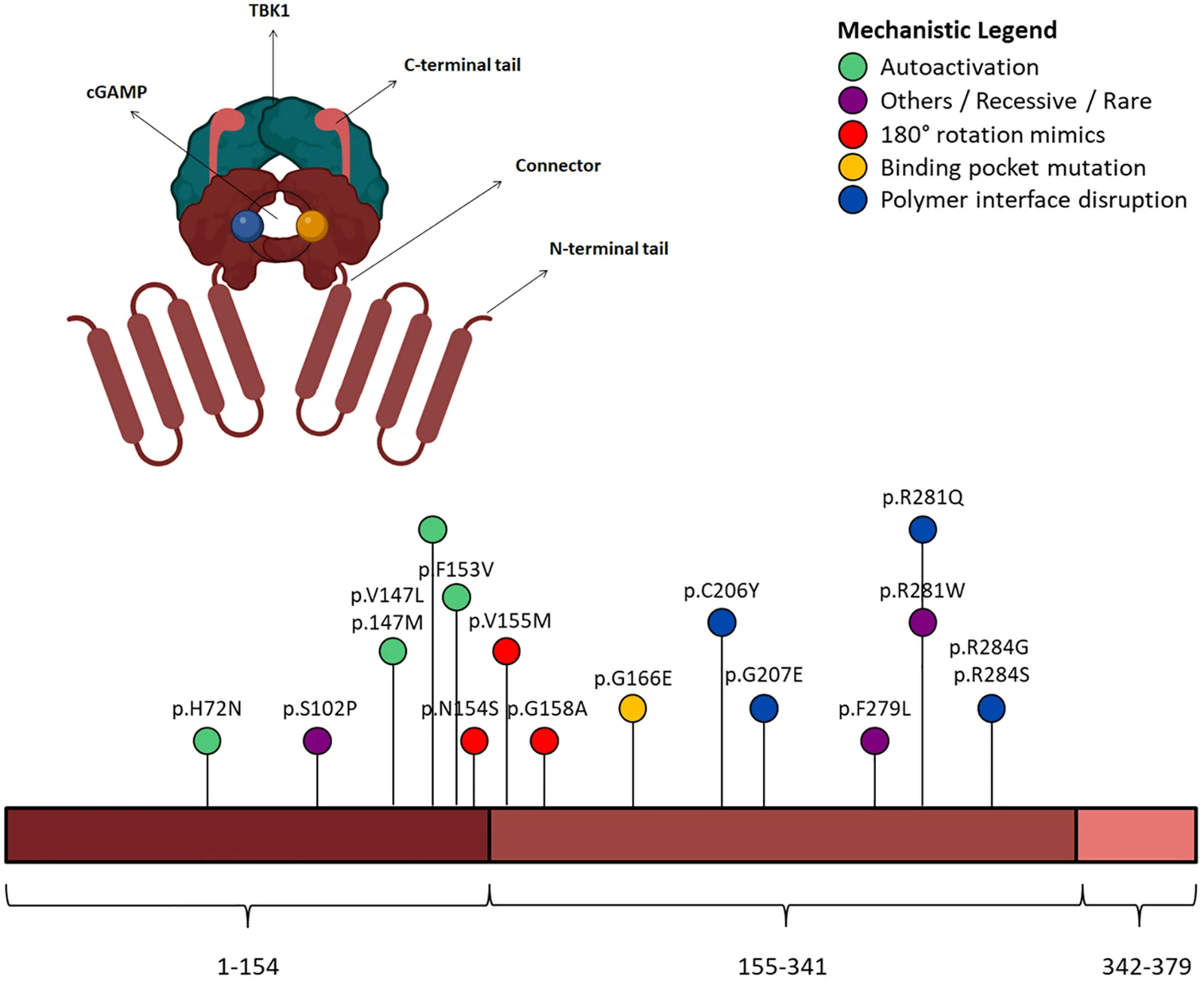
Mutational landscape of STING1 in SAVI. Top Panel: 3D structural model of the STING1 dimer, highlighting key functional regions including the transmembrane domains, the connector helix, the cGAMP binding pocket, and the C-terminal tail responsible for TBK1 recruitment. Bottom Panel: Linear protein representation (residues 1-379) mapped with SAVI-associated mutations. the human STING protein consists of an N-terminal region (aa 1–154) containing four transmembrane domains, and a C-terminal domain (CTD, aa 155–379). The CTD includes the dimerization domain (aa 155–341), where the majority of SAVI-associated mutations are clustered, and the carboxy-terminal tail (CTT, aa 342–379), which is essential for the recruitment of downstream signaling factors such as TBK1. Mutations are represented by colored flags and categorized into five classes based on their molecular impact.

*STING1* encodes a transmembrane ER protein involved in IFN production (**Figure 2**). The human STING protein (amino acids 1–379) contains four transmembrane domains (TMs) and a C-terminal domain (CTD, amino acids 138–379), which encompasses the dimerization domain (DD) and the carboxy-terminal tail (CTT, amino acids 342–379) (41). STING forms a symmetrical, butterfly-shaped dimer with a deep cleft at the interface between the two protomers, stabilized by hydrophobic interactions (41, 42). Physiologically, STING1 is activated by binding to cyclic GMP-AMP (cGAMP), a second messenger synthesized by cGMP-AMP synthase (cGAS) upon recognition of cytoplasmic dsDNA.

### 3.2 Molecular consequences of gain-of-function mutations and canonical signaling

In SAVI heterozygous gain-of-function (GOF) mutations cause constitutive STING activation independent of cGAMP binding, leading to continuous, unregulated trafficking between the ER and the ERGIC through two main pathways: STING- canonical -dependent Type I interferon activation and STING-dependent Type I interferon-independent pathway (28).

The canonical pathway involves the translocation of constitutively active STING to the ERGIC and the Golgi apparatus. Here, STING recruits and activates TBK1, which in turn phosphorylates IRF3. Phosphorylated IRF3 then translocates to the nucleus to induce the massive transcription of IFN-β and other interferon-stimulated genes (ISGs). This autocrine and paracrine loop sustains the high “interferon signature” typical of SAVI patients.

### 3.3 IFN-independent pathways: cellular stress, regulated cell death and inflammasome activation

The IFN-independent pathways activated by STING contribute significantly to the vasculopathy and systemic inflammation likely through TNFα, IL-6 and IL-21 cytokines. Beyond the canonical transcription of TI IFN, hyperactive STING variants trigger profound non-canonical cellular responses (**Figure 1**). Mutant STING induces severe ER stress and alters cellular homeostasis (43). Concurrently, aberrant STING activation modulates the autophagy machinery though the mTOR-AMPK-ULK1 axis, recruiting WIPI2 and LC3, contributing to metabolic exhaustion and tissue damage in target organs (43–45).

Mutant STING signaling engages interferon-independent programmed cell death pathways. This includes the IRF3-BAX induction of apoptosis (46), alongside necroptosis mediated by TNFR-RIPK1/RIPK3/MLKL axis (47). These non-canonical pathways directly drive heightened apoptosis in lymphocytes, explaining the severe cellular depletion and progressive immune dysregulation characteristic of the syndrome.

Furthermore, sustained STING activation stimulates innate immune responses through NLRP3 inflammasome assembly. The downstream recruitment of ASC, caspase-1, and NEK7 leads to the proteolytic maturation of pro-IL-1β and pro-IL-18, coupled with gasdermin D (GSDMD) cleavage and subsequent pyroptosis (48).

These IFN-independent effects, along with a secondary induction of Type II IFN (IFN-γ) signaling, may explain why JAK inhibitors (JAKi) often fail to fully prevent interstitial lung disease (ILD) progression and obtain a complete remission of the cutaneous phenotype in SAVI individuals (49).

## 4 STING1-related dominant SAVI

### 4.1 Clinical features and systemic manifestations

The main clinical features of SAVI include: systemic involvement with intermittent low-grade fever, malaise, chronic anemia, failure to thrive, cutaneous involvement and pulmonary interstitial disease (**Supplementary Table 1**) (12, 50–54). The disease has an early onset, in the first weeks of life, rarely after 1 year of age.

### 4.2 Cutaneous manifestations and mucosal involvement

The first clinical manifestations are generally cutaneous lesions, that typically affect the face, with a papulo-follicular appearance, and the extremities (fingers, ears, tip of the nose), where they manifest with a wide variability of presentation: erythematous or purpuric plaques, nodules, livedo reticularis, or very painful ulcerative lesions that evolve into eschars with tissue loss, up to digital amputation on a vasculitic basis. Cutaneous manifestations typically worsen after exposure to cold (12, 50–54). Mucosal involvement may also be present with manifestations ranging from aphthosis and oral ulcerations to nasal septum perforation. Periungual erythema and onychodystrophy are also commonly observed, which could represent prodromes of the pathology. Another expression of cutaneous involvement, found in some affected individuals, is Raynaud’s phenomenon, which, when investigated by capillaroscopy, shows tortuosity of the nailfold capillaries, in the absence of a clear scleroderma pattern.

### 4.3 Pulmonary involvement and interstitial lung disease

The other most characteristic clinical aspect of SAVI syndrome is pulmonary involvement: it consists of the development of interstitial lung disease (ILD), initially asymptomatic, which, over time, evolves toward pulmonary fibrosis. The most frequently reported symptoms are the association of cough and tachypnea, up to dyspnea on exertion or at rest, with the need for oxygen supplementation in the most severe cases (12, 50–54).

### 4.4 Histopathological findings

On the histopathological point of view, skin biopsies typically reveal a vasculopathy, most commonly presenting as leukocytoclastic vasculitis characterized by neutrophil infiltrates and microthrombotic angiopathy of small dermal vessels; however, perivascular inflammatory infiltrates and nodular granulomatous dermatitis have also been described (53–55). Lung biopsies mainly demonstrate interstitial fibrosis and chronic inflammatory changes, including lymphoid follicles with germinal center organization or non-specific mixed B-cell and T-cell infiltrates; in some cases, pulmonary hemorrhagic vasculitis and desquamative interstitial pneumonia have also been reported (56).

## 5 Expanding the genetic landscape of SAVI

### 5.1 *STING1*-related recessive SAVI

Traditionally, SAVI has been associated with dominant GOF mutations in *STING1*, but recent studies have also identified cases where recessive mutations in this same gene have led to a pathology with overlapping clinical manifestations to SAVI. In these cases, both copies of the *STING1* gene carry mutations (homozygosity or compound heterozygosity), which can alter protein function in ways different from dominant mutations. This can still lead to abnormal activation of the immune system and to a clinical picture similar to SAVI (**Supplementary Table 2**).

Lin et al. identified 6 patients from 4 unrelated families, all of Arab ethnicity, carrying pathogenic STING1 variants p.R281W that caused the disease only in homozygosity. The patients presented clinical manifestations overlapping with SAVI, with cutaneous (maculopapular rash, vasculitis) and pulmonary (diffuse parenchymal opacities) involvement (57).

Moreover, Alghamdi et al. described two siblings carrying a homozygous variant in the *STING1* gene that led to constitutive activation of the STING and the SAVI phenotype. Whole Exome sequencing (WES) revealed a novel homozygous variant NM_198282.3: c.841C>T (p.R281W) in exon 7 of the *STING1* gene. Computational structural analysis of the mutants revealed changes in the structure of the STING protein. Elevated serum levels of IFN-β were observed in patients compared to control family members. Treatment with ruxolitinib suppressed the inflammatory process, decreased IFN-β levels and reduced disease progression (58).

Further supporting these findings, Wan et al. also described the presence of recessive *STING1* mutations and clinical manifestations consistent with SAVI (59).

The existence of recessive forms of SAVI caused by mutations in *STING1* broadens the spectrum of pathologies associated with this gene and highlights the importance of also considering this possibility in the differential diagnosis of patients with symptoms suggestive of SAVI, especially in the presence of consanguinity or a family history of autoimmune diseases.

### 5.2 Asymptomatic SAVI individuals and the protective modulatory role of common *STING1* alleles

Recent clinical and genetic discoveries have identified healthy, asymptomatic individuals carrying classical SAVI-driving mutations (such as p.V155M or p.R281Q) who co-inherit common human *STING1* variants, specifically the AQ (G230A-R293Q) allele. Mechanistic insights from pre-clinical models demonstrate that while these compound genotypes may still exhibit TI-IFN pathway activation, the presence of residue 293 alterations (characteristic of AQ and HAQ alleles) confers a resistance to STING1-induced lymphocyte cell death (60). Consequently, this prevents the severe depletion of regulatory T cells (T-regs) and CD4+ T cellpenia typically driving SAVI immunopathology. This modulatory phenomenon explains the existence of a broader, yet underrecognized, spectrum of incomplete penetrance and asymptomatic carriers in the general population, highlighting the critical need to interpret *STING1* variants within the wider context of human genetic heterogeneity.

## 6 A mutational perspective of STING1 activation mechanisms in SAVI

### 6.1 Mutation clusters and structural mechanisms of activation

While in SAVI a direct genotype-phenotype association is not evident, differences in clinical presentation have been associated with mutations occurring in different regions of *STING1*, specifically exon 5 (first mutation cluster) and exons 6 and 7 (second mutation cluster) (61, 62).

The most common STING1 variants (p.V147M, p.N154S and p.V155M) are located in the same mutation cluster (connector helix loop) and are thought to trigger the constitutive activation of STING1 by inducing a 180° rotation in the ligand-binding domain that consequently leads to STING1 oligomerization (63). This activation process occurs independently of any interaction with its ligand cGAMP. Other substitutions within the second mutation cluster (p.C206, p.R281, and p.R284) are located in the polymerization interface and are assumed to inhibit the auto-suppression of STING1 oligomerization (29, 63, 64).

In a recent systematic review, Dai et al. compared the clinical features of patients with p.N154S and p.V155M mutations: patients with the p.N154S mutation exhibited earlier disease onset and more severe cutaneous lesions compared to those with the p.V155M mutation, while there was no significant difference in respiratory manifestations (65). More recently, Liu et al. reported three novel SAVI-causing mutations (p.H72N, p.G158A, p.F153V), identifying p.H72N, as the first disease-causing GOF-mutation in the transmembrane linker region of STING (57).

These observations led to the development of an adjusted model of STING1 activation that accommodates all currently reported STING1-causing mutations in exons 3, 5, 6 and 7 (57). STING1 is kept inactive by the C-terminal tail blocking of the polymer interface, which prevents polymerization through a side-by-side packing. Ligand binding induces 180° rotation of the ligand binding domain and leads to a conformational change in the polymer interface, which results in loss of C-terminal tail binding. This clears the polymer interface and allows polymer formation via side-by-side packing.

### 6.2 Classification of SAVI-causing mutations

SAVI-causing mutations can be categorized into 4 classes based on their effects on STING1 protein structure and activation (**Figure 2**).

1. Rotated conformation mimics: mutations p.G158A, and possibly p.N154S and p.V155M, located in the tightly packed connector region, promote a rotated conformation of STING1, mimicking the active, ligand-bound dimer.
2. Polymer interface disruption: the second class, involving residues R281, R284, C206, and G207 at the polymer interface, directly disrupts binding to the autoinhibitory C-terminal tail. This allows side-by-side packing of STING1 without the typical 180° rotation.
3. Constraint relief and autoactivation: mutations p.H72N, p.F153V, p.V147L, and p.V147M constitute the third class. These mutations relieve constraints on the polymer interface, leading to autoactivation of STING1 without requiring rotation. Specifically:
4. H72 and V147, located in the supporting arm that stabilizes the ligand-binding head, normally maintain C-terminal tail binding to the polymer interface. Mutations at these sites disrupt this interaction.
5. F153, positioned directly beneath the polymer interface, normally restricts its movement and prevents STING1 polymerization. The p.F153V mutation, by replacing the larger phenylalanine with the smaller valine, is predicted to increase the flexibility of the polymer interface, weakening C-terminal tail binding and thus relieving autoinhibition.
6. Ligand-binding pocket mutation: the fourth class consists of the p.G166E mutation, located in the ligand-binding pocket. This mutation results in very low-level autoactivation and also abolishes responsiveness to cGAMP. Current models do not fully explain this autoactivation, and it is unclear whether this low level alone is sufficient to cause SAVI. Further research is needed to determine the proper mechanism of autoactivation in this context and the potential role of other ligands in the development of disease.

## 7 Toward precision medicine in SAVI

### 7.1 Efficacy and safety profile of JAKi

#### 7.1.1 Clinical outcomes and challenges

Currently, no validated therapeutic protocols exist for effective disease control. While patients described in the literature have received high-dose corticosteroids and intravenous immunoglobulins (IVIG), during acute exacerbations, treatments with methotrexate, mycophenolate mofetil, hydroxychloroquine, infliximab, and rituximab have yielded unsatisfactory results. Notably, some patients treated with JAKi have demonstrated favorable clinical responses, enabling the discontinuation of steroid therapy (39, 66, 67). Fremond et al. (39), reported 17 patients treated with JAKi, allowing rapid clearance of fever, heterogeneous improvement of lung, and skin diseases depending on severity and damage. The best response was observed with ruxolitinib, a JAK1/2i, in patients treated early before the occurrence of irreversible damage especially to the lung, underlying the importance of early diagnosis (39). Tolerance was overall good, but the risk of viral respiratory infections in patients with poor lung function deserves particular attention (39). Of note, Sanchez et al. (67) reported a BK viremia in half of the patients with interferonopathies treated with baricitinib, with one patient developing renal failure due to interstitial nephritis, a complication seen in only 1 patient in Fremond’s cohort (68). We observed similar encouraging results with ruxolitinib (69) in three children aged 3–13y, with cutaneous and pulmonary involvement associated with recurrent fever and growth failure and renal involvement in one child. All patients were able to taper glucocorticoids. However, one patient displayed multiple severe viral infections after starting therapy, suggesting that these drugs may significantly increase this risk (69). Tofacitinib significantly improved skin lesions in a 9y-old child with SAVI, but did not ameliorate pulmonary involvement after two months of treatment. This was attributed to already established lung damage (70). In another case report, Balci et al. (71) described a 6-month-old infant who failed ruxolitinib therapy but subsequently responded to baricitinib.

#### 7.1.2 Genotype-dependent responses

Responses to JAKi are not universal and appear genotype-dependent. While some patients show promising responses to JAKi, *in vitro* studies suggest that these drugs may not be effective for all mutations. Specifically, the JAKi tofacitinib fails to inhibit dsDNA-triggered, STING-dependent IRF3 phosphorylation in cells expressing the p.R284S mutation (72). Accordingly, we reported worsening of ILD despite initial transient improvement in a patient with the p.R281Q mutation (69). Similarly, in the Lin et al. report (57), patients receiving JAKi showed divergent outcomes: one patient achieved clinical improvement with continued oxygen dependence, while the other patient died of respiratory failure despite steroid therapy and short-term treatment with tofacitinib. These findings suggest that mutations affecting residues R281 and R284 may predict suboptimal responses to JAKi, highlighting the need for genetic profiling to guide treatment decisions.

#### 7.1.3 Alternative molecular-axes

Beyond the JAK/STAT cascade, alternative molecular axes are being explored. It has been reported recently that the stimulation of AMPK activity releases ULK1-mediated phosphorylation of STING1, which inhibits STING ability to phosphorylate IRF3. Therefore, ULK1 regulators may be an alternative therapeutic option for STING-induced inflammatory diseases, which warrants further investigation (72).

Interestingly, as reported in **Supplementary Table 3**, the existence of *STING1* heterogeneity among ethnicities—reflecting historical evolutionary selections such as the positive selection of the HAQ allele during the Out-of-Africa migration—underlines the importance of considering naturally occurring *STING* variants and their distinct functional consequences (both Type I IFN-dependent and independent pathways) for both association with disease and pharmacological drug design at the same time (73). Recently Alehashemi et al. (74), showed the efficacy of anti-IFNAR1 antibody (anifrolumab) with durably suppressed IFN scores, in a patient with SAVI who had vasculitic ulcers and systemic inflammation refractory to JAKi (baricitinib) associated with tocilizumab. In another recent paper Kretzschmar et al. (75), showed in four patients with SAVI that anifrolumab exerted an additive effect over JAKi (baricitinib) alone. In two of these patients, monotherapy with anifrolumab is sufficient to retain a suppressed IFN-I signature and clinical improvement (75). Despite promising results, only these few anecdotal studies show the efficacy of anifrolumab therapy in SAVI patients, and only in two patients as monotherapy. Furthermore, long-term efficacy and safety data on this treatment are lacking.

### 7.2 Limitations of JAKi therapy in SAVI

Despite the growing adoption of JAKi (such as ruxolitinib, baricitinib, and tofacitinib) as the primary targeted therapy for SAVI, the overall quality of clinical evidence remains extremely low, accounting predominantly for isolated case reports, small familial series and retrospective multicenter registries (Evidence level IV according to the Oxford Centre for Evidence-Based Medicine classification - OCEBM). Worldwide collections comprise 21–51 published patients, with a restricted subset of individuals receiving targeted JAKi therapy under highly heterogeneous dosing regimens and schedules (55).

Furthermore, clinical outcomes reveal that while JAKi consistently induce rapid and robust improvements in systemic fevers, arthralgia, and acute cutaneous ulcers, their impact on established ILD is markedly limited (76). Patients who have already developed structural pulmonary fibrosis frequently experience disease progression and respiratory failure despite optimal JAKi. This lack of response is also mutation-dependent; notably, specific genotypes—such as families carrying homozygous R281W mutations—display complete non-responsiveness to ruxolitinib regardless of cutaneous control (59). Consequently, the debate on whether emerging mechanism-based biologics—such as anti-human IFNAR1 monoclonal antibodies (e.g., anifrolumab)—will eventually replace JAKi as the first-line treatment remains open, particularly to overcome the downstream limitations observed with upstream kinase inhibition.

Finally, published observational windows remain short and variable, averaging between 2.1-2.6 years in larger cohorts, limiting the comprehension of late-stage pulmonary flares, or long-term cumulative toxicities in developing pediatric populations (76). In addition, the widespread use of concurrent or shifting background treatments—including high-dose systemic corticosteroids, methylprednisolone, IVIG, and cytotoxic agents—introduces significant confounding bias, making it challenging to isolate the precise therapeutic efficacy attributable solely to JAKi.

### 7.3 Toward a precision framework: integrating quantitative synthesis, genotype-driven therapy and genetic modifiers

Analyzing the pooled spectrum of reported -pediatric and -adult cohorts distinct genetic and phenotypic architectures emerged (**Supplementary Table 4**): dominant GOF alleles account for the vast majority of cases (~80–85%, driven by frequent residues such as V155M and N154S), while recessive forms represent roughly 15–20%. Clinically, this genetic landscape translates into an overwhelming burden of progressive ILD (affecting >85–90% of symptomatic individuals across both inheritance patterns) and severe cutaneous vasculopathy (~80%), frequently accompanied by a complex immunological profile characterized by high rates of ANA (~50–60%) and ANCA (~30%) positivity alongside profound cellular defects (e.g., CD4+/CD8+ and Treg depletion).

Building upon this clinical baseline, therapeutic management with JAKi demonstrates variable efficacy—ranging from partial cutaneous and pulmonary stabilization to primary refractoriness. Pharmacological response is largely independent of the mode of inheritance but heavily reliant on early intervention before irreversible structural damage occurs, alongside specific genotype-dependent limitations observed in high-risk variants (e.g., V155M, N154S, R281W). Consequently, advanced disease stages necessitate high-risk surgical and cellular interventions. End-stage management highlights the critical need of bilateral lung transplantation (BLT) and allogeneic hematopoietic stem cell transplantation (allo-HSCT) to correct the hematopoietic compartment, though post-transplant courses remain heavily challenged by viral reactivations (such as BKV) and severe infections.

Finally, the clinical penetrance of these pathogenic variants is further modulated by human genetic heterogeneity. The existence of modified asymptomatic carriers and protective co-inherited alleles demonstrates how genetic background could profoundly influence phenotypic severity by rescuing immune cells from STING-induced cytotoxicity. Together, these quantitative and mechanistic insights—spanning from epidemiological distributions and autoantibody profiles to genotype-phenotype correlations and advanced cellular therapies—emphasize that future management of SAVI must move beyond a one-size-fits-all approach toward an individualized, multi-layered precision medicine strategy.

### 7.4 Comparative spectrum and therapeutic management of pulmonary involvement in SAVI vs. TI-IFNs

ILD represents a hallmark, life-threatening manifestation of SAVI and related disorders such as COPA syndrome, driving progressive respiratory failure and significant morbidity. As outlined in **Supplementary Table 5**, this prominent pulmonary involvement marks a critical clinical divergence from other monogenic TI-IFNs—this prominent pulmonary involvement marks a critical clinical divergence from other monogenic type I interferonopathies (TI-IFNs)—most notably Aicardi–Goutières syndrome (AGS), CANDLE (Chronic Atypical Neutrophil Dermatosis with Lipodystrophy and Elevated Temperature) syndrome, USP18 deficiency, and STAT2 gain-of-function—which characteristically lack prominent pulmonary manifestations and ILD. Consequently, while neurological impairment dictates the clinical trajectory of AGS and systemic multi-organ autoinflammation characterizes CANDLE, therapeutic interventions such as BLT are uniquely restricted to end-stage pulmonary failure in SAVI and COPA syndrome.

However, because traditional immunosuppression yields limited pulmonary benefit and JAKi frequently fail to reverse established structural fibrosis, LT remains the ultimate life-saving option for selected refractory cases. Patient selection demands careful perioperative management, accounting for systemic microvascular fragility, baseline immune dysregulation, and high susceptibility to post-transplant infections. Unlike solid-organ transplants that replace the fibrotic tissue sink without correcting the systemic genetic defect, allo-HSCT addresses the hematopoietic immune compartment, reflecting the dual pathogenetic contribution of both immune and stromal compartments in SAVI.

### 7.5 Transplants and advanced therapies

#### 7.5.1 Transplantation challenges

Given the limitations of current pharmacological therapies, researchers are exploring alternative approaches, including allo-HSCT and HSC gene editing (GE) based-therapy. While allo-HSCT can eliminate autoinflammatory immune cells, it faces unique biological hurdles in the context of SAVI. Challenges include the availability of HLA-matched donors, delayed reconstitution, and the risk of graft-versus-host disease (GvHD). Furthermore, allo-HSCT does not address the extra-hematopoietic defects caused by mutant STING1, which is highly expressed in lung stromal cells, including bronchial epithelial cells and type 2 pneumocytes. Experimental models (Rag1^−/−^ STING^N153S^) suggest that while T-cells are necessary for full disease expression, the non-hematopoietic compartment plays a significant role. On the other side, the hematopoietic role has been demonstrated by two cases of disease recurrence on grafted organ, lung and liver. The first case describes the recurrence of the initial respiratory manifestation (intra-alveolar hemorrhage) on transplanted lungs in a 17-year-old girl whose diagnosis of SAVI was made after lung transplants (LT) (77). The second case is about a 1-year-old girl who underwent a liver transplantation for multiple biliary cysts that recurred 2 years after without any other identifiable etiology (78). Taken together, these clinical observations define SAVI as a systemic, whole-organism disease. This perspective implies that transplantation of solid organ may not be curative if the systemic disease is not controlled with immune suppression or targeted therapies.

#### 7.5.2 Clinical experience with lung transplantation

A total of 7 patients have been reported (**Supplementary Table 6**) to have underwent LT for SAVI-related end-stage lung disease, accounting for 8 transplant procedures (one patient underwent a second LT) (12). The patients ranged in age from 13.9 to 34 years at the time of first LT. All but one procedure (Clarke et al.) were bilateral lung transplants (BLT). The most commonly identified STING1 variant was the V155M substitution (c.463G>A), documented in 6 of 7 patients; the remaining patient (Lv et al.) carried a novel c.842G>A variant (p.R281Q). Six of seven cases were pediatric or young adult; the case reported by Lv et al. represents the first detailed report of successful bilateral LT in a 28-year-old adult with adult-onset SAVI and a 5-month post-operative follow-up (79). A critical issue emerging from the review is whether the diagnosis of SAVI was established before or after LT, as this directly determines the possibility of implementing targeted pre- and peri-operative therapeutic strategies. In the three earliest reported cases (77, 80, 81), the diagnosis of SAVI was established only after transplantation, preventing use of targeted therapies (39). The three patients of Berrada et al. (82) and the patient of Lv et al. (79) received their genetic diagnosis prior to LT. All three patients had been started on ruxolitinib before LT as off-label treatment, with transient or partial clinical improvement. Despite ruxolitinib, respiratory function continued to deteriorate in all three, eventually meeting LT criteria. The patient reported by Lv et al. received his diagnosis by clinical WES performed during hospitalization. However, no JAKi was started before LT, given the rapid clinical deterioration and the heterogeneous evidence supporting pre-operative JAKi in adults with SAVI.

A key observation from Berrada et al. was that despite continued ruxolitinib administration, the IFN signature (measured as median fold-change of ISG mRNA expression) did not normalize in any of the three patients post-LT. Large fluctuations without sustained reduction below the lower limit of normality were observed in P1 over 2.6 years of follow-up. Post-transplant mortality in the reviewed cohort is substantial: 4 of 7 patients (57%) died following LT. The causes of death were heterogeneous but consistently reflected complex interplay between immunological dysregulation intrinsic to SAVI, immunosuppression-related complications, and surgical/procedural sequelae. Notably, the two longest survivors (Clarke, 8 years; Berrada P1, 2.6 years and ongoing) both demonstrate that meaningful medium-term survival is achievable. However, among the deaths, two occurred within the first year (3 months and 33 days), and one at 4 years from a delayed complication (bronchial wall rupture). These findings highlight that the post-transplant risk in SAVI is not confined to the peri-operative period but extends over the entire follow-up horizon. Complications that appear to be specific to SAVI include thromboembolic events (TE), reported in all patients by Berrada et al. at 9–17 months post LT, possibly related to chronic systemic inflammation and JAKi use; airway and respiratory complications, in 2/3 patients by Berrada et al. with a bilateral bronchial stenosis requiring stenting and tissue granulation requiring interventional procedure and ultimately ending with bronchial wall rupture and death at 4 years post-transplant; infections including BK virus, acyclovir-resistant varicella-zoster virus (VZV), post-transplant lymphoproliferative disorder (PTLD), and fungal infections.

#### 7.5.3 Future perspectives and allo-HSCT

A recent report described a successful allo-HSCT in a SAVI patient harboring the p.N154S mutation with severe pulmonary and cutaneous involvement refractory to multiple JAKi. The patient was transplanted from a matched sibling donor following myeloablative conditioning with busulfan, cyclophosphamide, fludarabine and anti-thymocyte globulin. The authors report only autoimmune hemolytic anemia as a complication, that responded to steroid. At 12 months post transplantation, the patient showed no signs of lung disease progression, resolution of skin disease, normalization of blood abnormalities and systemic inflammation (83). This report is of particular interest since it provides evidence that the procedure is potentially curative opening the possibility also to hematopoietic targeted gene therapy approach. However, given the high morbidity associated with current transplantation protocols (84), the successful outcome of this allo-HSCT also highlights the therapeutic window required for future GE applications (85, 86). Specifically, in the absence of a perfectly HLA-matched donor, or in patients with advanced organ failure, pharmacological bridging with JAKi would be essential to achieve immune stabilization prior to any curative genetic intervention (87, 88). This sequential approach—utilizing JAKi to dampen the systemic interferon storm followed by targeted hematopoietic correction—may offer the safest path forward to improve the dismal prognosis associated with traditional transplantation (89, 90). Moreover, this successful human allo-HSCT outcome strongly supports a pathogenetic model where hematopoietic cells—specifically T cells and monocytes—act as major drivers of SAVI pulmonary and systemic immunopathology (39). While this aligns directly with clinical recurrences on solid-organ grafts and several murine lines, it contrasts with complementary experimental models (such as endothelial-restricted mutant STING expression), underscoring that both hematopoietic and non-hematopoietic compartments cooperatively shape the complex clinical architecture of SAVI (91).

## 8 Biomarkers and precision medicine in TI-IFN

### 8.1 ISG score, diagnostic utility and clinical monitoring

The paradigm of precision medicine relies heavily on the identification and validation of robust biomarkers capable of guiding early diagnosis, patient stratification, and targeted therapeutic monitoring. In the context of TI-IFN and related inflammatory disorders, the direct quantification of circulating Type I IFNs in plasma or serum remains technically challenging due to their potent biological activity at concentrations below the threshold of standard assays. To overcome this limitation, the TI-IFN signature—defined by the upregulated expression of Interferon-Stimulated Genes (ISGs)—has emerged as a reliable and sensitive surrogate biomarker of IFN-driven inflammation.

Seminal studies, such as the case-control cohort analysis by Rice et al., demonstrated that robust IFN-signatures comprising core ISGs (such as *IFI27, IFI44L, IFIT1, ISG15, RSAD2*, and *SIGLEC1*) were consistently present across monogenic interferonopathies. While initially characterized extensively in conditions like AGS, this persistent transcriptional signature is equally hallmark to other autoinflammatory spectrums, including SAVI (92).

Traditional RT-qPCR methods relying on variable biological healthy donors introduce inter-assay and inter-center inconsistencies. Consequently, recent methodological advancements have introduced standardized quantification strategies using synthetic controls with plasmid-based standard curves that successfully eliminate inter-laboratory variability, normalize gene scores through stable housekeeping references as *HPRT1* and *TBP*, and establish robust diagnostic cut-offs, which facilitates multi-center data sharing and trial harmonization (93). Furthermore, the IFN signature is crucial in differentiating monogenic interferonopathies, juvenile systemic lupus erythematosus, and active juvenile dermatomyositis from autoinflammatory conditions driven by other cytokine pathways such as periodic fevers and juvenile idiopathic arthritis, which characteristically display normal or negative interferon scores (94). Moreover, clinical evidence demonstrates that administration of anifrolumab successfully achieves sustained suppression and normalization of elevated IFN signature genes—overcoming the suboptimal molecular responses occasionally observed with upstream kinase inhibition alone—and that this active molecular reversal correlates directly with robust clinical improvements in refractory SAVI patients (95).

Despite its high diagnostic sensitivity, a critical limitation in conditions like SAVI is that the magnitude of the blood-derived Type I IFN signature often shows a poor linear correlation with pulmonary disease severity (such as ILD) or exact clinical responses to JAKi. Thus, relying solely on the core ISG score is insufficient for comprehensive prognostic monitoring.

### 8.2 Secondary molecular and cellular markers

To address the limitations of the core IFN signature, recent literature highlights the role of secondary IFN-induced chemokines and pro-inflammatory cytokines as dynamic surrogate markers. CXCL10 (IP-10) and CXCL9 are IFNγ-inducible chemokines markedly elevated in SAVI and related disorders, showing a much tighter correlation with systemic disease activity, cutaneous lesions, and progression of pulmonary fibrosis than the baseline ISG score alone (96, 97). Elevated serum levels of IL-6, TNF-α, and markers of monocyte/macrophage activation mirror the severe systemic vasculopathy and tissue destruction characteristic of STING-mediated inflammation, serving as dynamic tools to monitor therapeutic efficacy during JAKi therapy (e.g., ruxolitinib, baricitinib) (98). Functional assays evaluating the Unfolded Protein Response (UPR) pathway also offer a direct molecular index of cellular pathogenetic load and tissue stress in SAVI patients (99, 100). Monitoring aberrant cell death profiles and CD8+ T-cell hyperactivation—which drives severe pulmonary and cutaneous inflammation—would also provide prognostic guidance to evaluate whether patients require escalation to advanced therapies. Finally, the integration of genetic screening through WES remains conclusive for definitive diagnosis and clinical management. These molecular profiles would significantly improve precision medicine—supporting the clinical evaluation of anti-interferon biological agents and JAKi shifting clinical management from purely symptomatic care to mechanism-based precision medical decisions.

## 9 Toward precision medicine in SAVI management

### 9.1 Comprehensive clinical management and decision-making algorithm in SAVI

**Figure 3:**
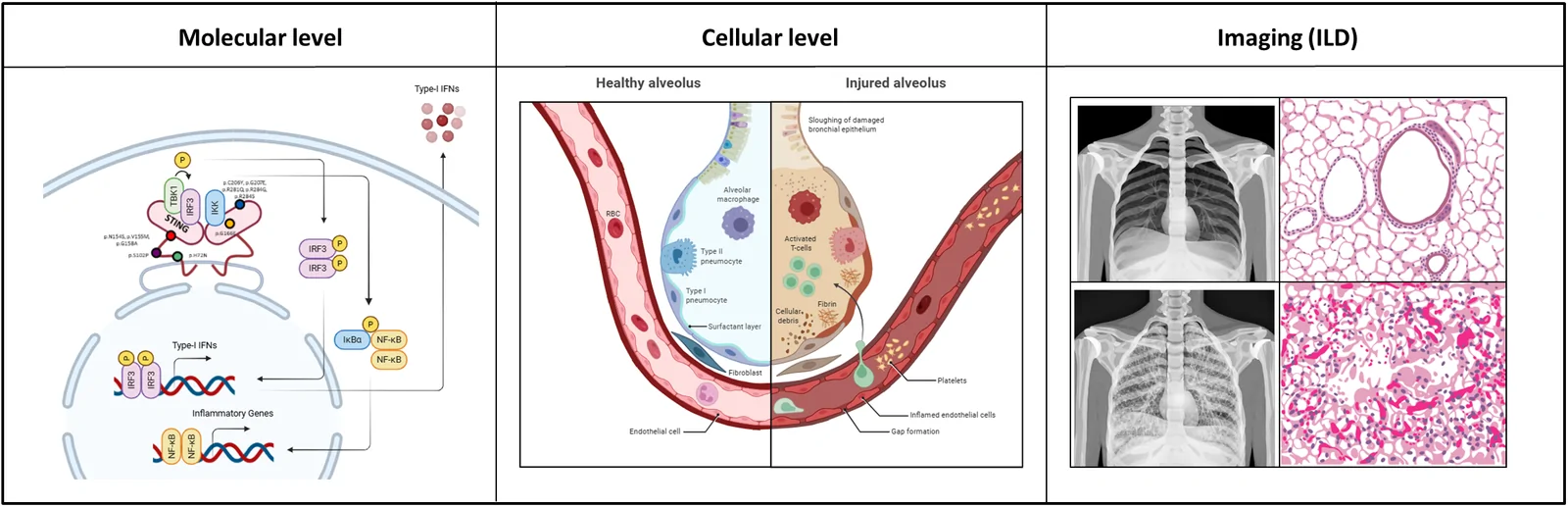
Schematic overview of the clinicopathological correlation in SAVI-associated ILD. The illustration links molecular drivers to tissue damage and radiological phenotypes across three progressive levels: molecular level, cellular level and histopathological level. This figure was created by BioRender.com.

**Figure 4:**
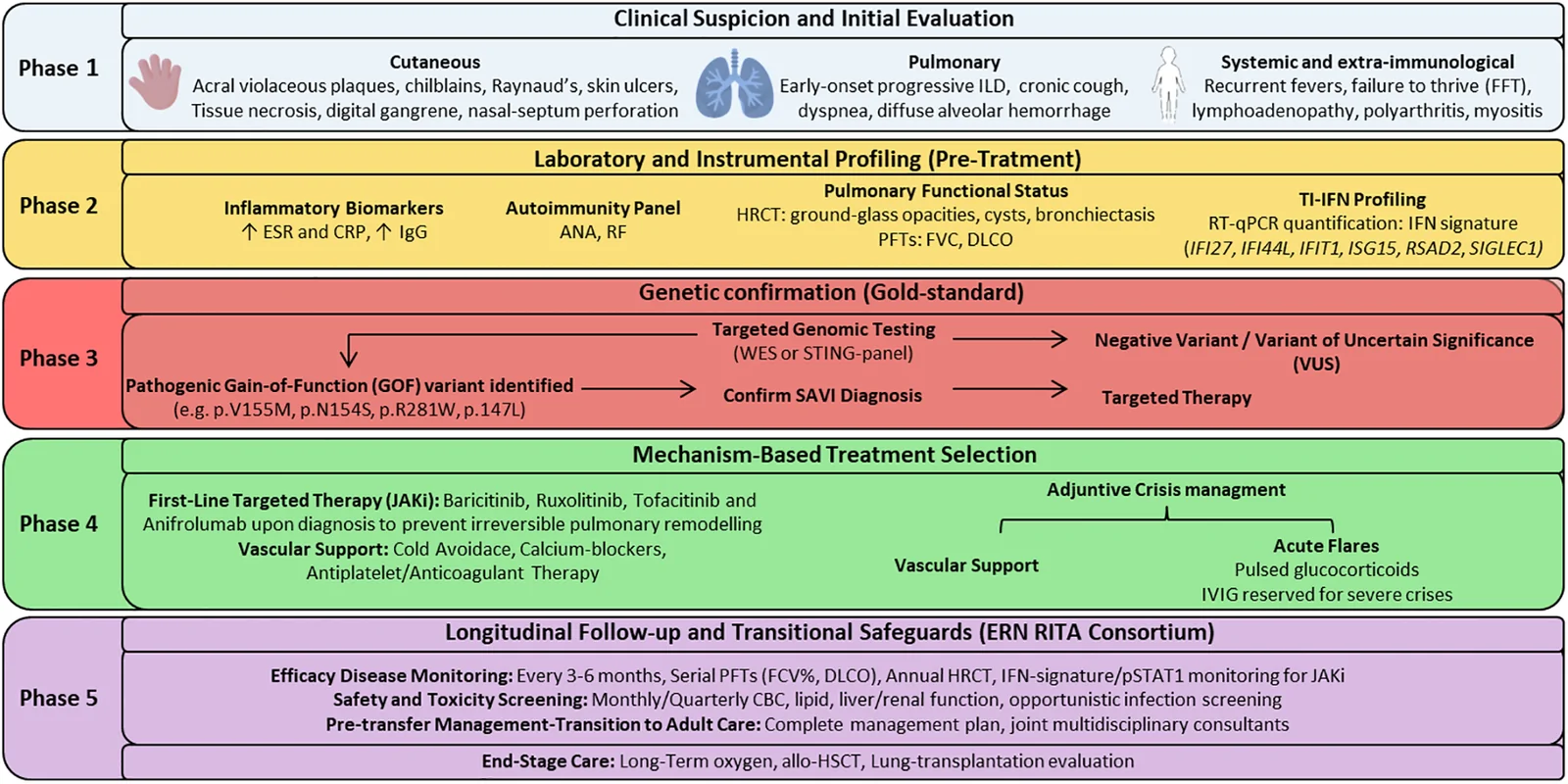
Comprehensive five-phase diagnostic, therapeutic, and transitional management algorithm for STING-associated vasculopathy with onset in infancy (SAVI). The framework is organized into five progressive clinical phases designed to guide clinical management.

To better understand the complex tissue damage underlying the disease, the clinicopathological correlation linking molecular STING hyperactivation to cellular injury and ILD is outlined in **Figure 3**. Building upon these pathophysiological foundations and translating recent molecular insights into structured clinical practice, patient management is further guided by a comprehensive five-phase diagnostic and therapeutic framework (**Figure 4**).

Following initial clinical recognition of the hallmark cutaneous, pulmonary, and systemic manifestations (Phase 1), patients undergo thorough laboratory, functional, and standardized type I interferon profiling (Phase 2). Definitive confirmation relies on targeted genetic testing of *STING1*, where identified GOF variants confirm diagnosis, while variants of uncertain significance prompt functional cellular assays (Phase 3). Therapeutic management centers on the immediate early initiation of targeted JAKi alongside vascular support and selective crisis management (Phase 4). Finally, longitudinal care implements regular multi-system monitoring, toxicity screening, structured transition safeguards aligned with the European Reference Network on Rare Immunodeficiency, Autoinflammatory and Autoimmune Diseases (ERN-RITA) Consortium Guidelines (101), and advanced interventions for end-stage complications (Phase 5). This patient-centered approach aims to optimize outcomes while minimizing treatment-related toxicity, ultimately improving the risk–benefit ratio for each individual patient.

Disease activity must be assessed at each visit using validated tools, including the SAVI Disease Activity Score (SDAS), a composite measure incorporating cutaneous, pulmonary, and systemic domains. The frequency of assessments should be stratified by disease severity: monthly for severe/refractory disease, quarterly for moderate disease, and biannually for mild/stable disease. Given the high morbidity associated with ILD in SAVI, spirometry and DLCO are required at baseline and every 3–6 months. High-resolution CT (HRCT) must be performed at baseline and annually, or earlier if clinically indicated. The use of quantitative CT scoring systems for fibrosis extent enhances sensitivity for detecting progression. Complete blood counts must be monitored weekly for the first month of JAKi therapy, then monthly, given the risk of cytopenias. Lymphocyte subset analysis and immunoglobulin levels are assessed every 3 months, particularly in pediatric patients, to evaluate for secondary immunodeficiency. Where available, measurement of JAKi plasma concentrations would help optimize dosing, particularly in pediatric patients where pharmacokinetic data are limited. Target trough concentrations remain to be established through future prospective studies.

### 9.2 Genotype-phenotype stratification

The specific *STING1* mutation can inform therapeutic decisions through several mechanisms. First, the nature of the amino acid substitution can influence the degree of constitutive STING activation; for example, the p.V155M and p.N154S variants are associated with stronger constitutive activation and more severe clinical phenotypes, potentially warranting earlier and more aggressive intervention with JAKi, whereas variants such as p.G166E may result in partial loss-of-function with a milder phenotype, where a watchful waiting approach with serial monitoring may be appropriate. Second, the location of the mutation within the STING protein may predict response to specific therapies, as mutations in the dimerization domain (e.g., V155M) may be more amenable to small-molecule inhibition of STING oligomerization, while mutations in the C-terminal tail (e.g., R284G) may preferentially benefit from downstream blockade of TBK1/IRF3 signaling—hypotheses that provide a rational framework for genotype-guided therapeutic decision-making despite requiring further experimental validation.

### 9.3 Therapeutic decision-making strategies

While dominant GOF mutations in STING1 represent 80–85% of cases (frequently affecting residues V155M and N154S) and recessive forms account for 15–20% (e.g., the homozygous p.R281W variant), treatment response to JAKi is not universal; critical mutations in residues R281 and R284 (such as p.R284S or p.R281Q) often exhibit primary resistance or poor clinical efficacy, leading to progressive interstitial lung disease (ILD) despite therapy. Third, the presence of additional genetic modifiers—such as polymorphisms in IFN regulatory genes, co-inherited variants in the type I IFN pathway, or protective co-inherited alleles like AQ/HAQ with alterations at residue 293—explains incomplete penetrance and the existence of asymptomatic healthy carriers by protecting T lymphocytes from STING-induced cytotoxicity, making comprehensive genetic testing like WES recommended at diagnosis to inform prognosis and guide therapy. Building on this stratification, clinical decision-making relies heavily on early intervention with JAKi (such as ruxolitinib and baricitinib) before irreversible structural lung damage occurs, while refractory cases or high-risk mutational profiles benefit from advanced therapies including anti-IFNAR1 monoclonal antibodies (anifrolumab) to durably suppress the interferon signature, bilateral lung transplantation (BLT) as a life-saving option for end-stage pulmonary complications despite significant post-transplant mortality, and allogeneic hematopoietic stem cell transplantation (allo-HSCT) to offer potential curative correction of the hematopoietic immune compartment.

### 9.4 Biomarker-driven surveillance

To monitor these interventions, biomarker-based surveillance must account for the limitations of the classic peripheral blood interferon signature (core ISG score), which often shows poor linear correlation with ILD severity or JAKi response, by utilizing dynamic secondary markers such as serum chemokines and cytokines (CXCL10/IP-10, CXCL9, IL-6, and TNF-α) that closely correlate with systemic disease activity, skin lesions, and pulmonary fibrosis progression, alongside routine functional evaluations via the SAVI Disease Activity Score (SDAS), spirometry with DLCO every 3–6 months, baseline/serial high-resolution computed tomography (HRCT), and strict monitoring of cytopenias and lymphocyte subsets to prevent opportunistic viral infections like BK virus.

## 10 Challenges and opportunities in SAVI research and treatment

### 10.1 Preclinical modeling: insights and limitations of murine phenotypes

The severity of SAVI symptoms and the constant threat of life-threatening complications highlight a significant unmet need for affected patients. Despite recent progress, several hurdles remain in fully understanding the disease pathogenesis and translating preclinical findings into bedside reality.

Investigating SAVI in animal models offers a promising approach to elucidate the disease pathogenesis and develop novel therapies. Several mouse models utilizing immunocompetent C57BL/6N mice engineered to express SAVI-associated mutations have demonstrated only partial recapitulation of the disease’s pathophysiology. These include knock-in mouse models harboring SAVI-associated mutations p.V154M or p.N153S (orthologues of the human p.V155M and p.N154S mutations), as well as mice expressing human STING. As summarized in **Supplementary Table 7**, these experimental models exhibit heterogeneous phenotypic severities and cellular involvements, providing a comprehensive comparative framework for their translational relevance to human SAVI.

#### 10.1.1 Cellular contributions and compartmentalization

Studies conducted in lethally irradiated p.V154M hosts reconstituted with wild-type hematopoietic stem cells (WT→VM chimeras) revealed that non-hematopoietic cells, particularly stromal cells, play a crucial role in initiating pulmonary inflammation by triggering and recruiting lymphocytes to the lung (102). However, subsequent work from the same group demonstrated that endothelial restricted expression of mutant STING is not sufficient to fully recapitulate the disease phenotype, indicating that coordinated activation of multiple cell types is required for complete disease manifestation (103). Notably, the p.V154M mutation drives a more severe phenotype compared to p.N153S, characterized by interstitial lung disease with inflammation and fibrosis, a hallmark clinical feature also observed in patients. This is accompanied by a combined immunodeficiency CID/SCID like phenotype that exceeds the severity of immune defects reported in humans (51, 52, 104). In addition, p.V154M mice display reduced survival compared with p.N153S models (51).

#### 10.1.2 Signaling pathways and mutation-specific outcomes

Further insights into disease mechanisms were obtained in knock-in mice carrying the p.N153S mutation in STING, in which lung pathology developed independently of cGAS, IRF3, and IFNAR1. This demonstrates that disease progression can occur by interferon-independent pathways downstream of STING activation, consistent with observations in p.V154M models (51, 52, 80, 105). Bone marrow transplantation experiments further demonstrated that several features of STING p.N153S associated pathology are intrinsic to the hematopoietic compartment (12, 105).

Interestingly, distinct results have been obtained in models expressing human STING (hSTING). In contrast to the p.N153S and p.V154M model, expression of constitutively active hSTING in hematopoietic cells leads to IFNAR1-dependent vasculopathy, indicating that disease manifestations in this setting rely on type I IFN signaling. In these mice, some clinical features reminiscent of the disease are observed, including systemic inflammation and vascular alterations; however, pulmonary involvement is limited and severe lung disease does not develop to the same extent reported in other SAVI models (106). These findings suggest that the cellular compartment driving pathology and the reliance on interferon signaling may vary depending on the specific STING mutation or experimental model.

Radiation chimera experiments also revealed mutation-specific disease outcomes: bone marrow from p.V154M mutant mice was able to transfer disease to wild-type hosts, whereas the p.N153S mutation was not. These studies further showed that T cell lymphopenia depends on T cell-intrinsic expression of the SAVI mutation (107).

Overall, these murine models recapitulate key clinical and immunological features of SAVI and demonstrate that pathological STING activation in both hematopoietic cells (including lymphoid and myeloid immune populations) and non-hematopoietic compartments, such as stromal and endothelial cells, contributes to disease development. Importantly, disease manifestations can arise through both interferon-dependent mechanisms, driven by signaling through the TI-IFN receptor IFNAR1, and interferon-independent pathways acting downstream of STING activation. Moreover, the distinct pathogenic effects of SAVI-associated mutations such as p.N153S and p.V154M highlight mutation-specific pathways that may contribute to the clinical heterogeneity observed in patients. Notably, models expressing hSTING seem to support a role for TI-IFN signaling in driving vasculopathy and systemic inflammation, providing complementary insight into the diversity of mechanisms underlying SAVI pathogenesis.

### 10.2 Novel therapeutic strategies and future molecular frontiers in SAVI

#### 10.2.1 Emerging non-genetic molecular therapies in SAVI

Beyond upstream kinase inhibition and cellular replacement strategies, several innovative non-genetic pharmacological approaches are currently under preclinical and clinical investigation to directly target dysregulated STING signaling pathways. As summarized in **Supplementary Table 8**, the evolving therapeutic landscape of non-genetic molecular therapies in SAVI encompasses several approaches, each characterized by distinct mechanisms, molecular targets, and clinical applicability. Small molecules specifically engineered to bind the cyclic dinucleotide (CDN) binding pocket or stabilize the inactive conformation of the STING protein hold significant promise (108). These compounds halt receptor oligomerization and downstream signal propagation regardless of the underlying GOF genotype, offering a direct mechanism to intercept pathological activation at the receptor level (109, 110).

The efficacy of these agents is deeply rooted in the structural pathology of SAVI mutations, such as V155M. In these cases, the bulky methionine substitution at the LBD α-helix forces the STING homodimer into a spontaneous 180° rotation and active oligomerization, effectively bypassing the upstream need for cGAMP (111). Consequently, while upstream cGAS inhibitors (such as VENT-03 or IMSB301) are rendered ineffective because the pathway is constitutively bypassed at the receptor level (112), advanced STING-targeted modalities have emerged as superior alternatives (113). Among these, TMD Allosteric Wedges (e.g., Y-320 class) act as molecular wedges within the ER lipid bilayer, mechanically preventing the stacking of transmembrane helices and arresting the ER-to-Golgi trafficking required for signaling, whereas Polymerization Blockers physically dismantle the signaling scaffold at the dimer-to-dimer interface, neutralizing V155M and other mutations with mutation-agnostic potency (114).

Targeting downstream signal transduction cascades has emerged as a viable alternative; pharmacological inhibition of TBK1 and its redundant homolog IκB kinase-epsilon (IKKϵ) uncouples STING activation from IRF3 and NF-κB responses (115). Furthermore, exploiting metabolic axes—such as the activation of AMPK—triggers ULK1-mediated phosphorylation of STING, effectively suppressing downstream signal output (116, 117).

Finally, RNA therapeutics, including allele-specific antisense oligonucleotides (ASOs) and small interfering RNAs (siRNAs), offer a tunable, reversible post-transcriptional mechanism to knock down hyperactive mutant *STING1* transcripts. These platforms bypass the permanent genotoxic risks associated with permanent GE while effectively reducing the cellular burden of the mutant protein (118).

#### 10.2.2 Advanced molecular platforms and future perspectives in SAVI

SAVI therapeutics extends toward advanced biological platforms and precision genetic correction as summarized in **Supplementary Table 9**. Another promising strategy involves using induced pluripotent stem cells (iPSCs) generated from SAVI patients (119–122). iPSCs can be differentiated into relevant innate immune cells or lung organoids providing a valuable platform to address critical knowledge gaps in SAVI pathology and evaluate novel therapeutic interventions (123). Concurrently, recent mechanistical insights using these models have refined our understanding of SAVI pathogenesis, specifically identifying radioresistant non-immune cell compartments, such as pulmonary endothelial and stromal cells, as the primary instigators of the fatal ILD phenotype (103, 124). This discovery confirms the absolute requirement for tissue-specific, *in vivo* delivery vehicles capable of penetrating structural parenchymal beds, rather than relying solely on hematological or *ex vivo* hematopoietic stem and progenitor cell (HSPC) reconstitution (97).

Furthermore, recent advancements in GE technology hold considerable therapeutic potential for SAVI treatments (125–127). The clustered regularly interspaced short palindromic repeats-associated protein 9 nuclease (CRISPR/Cas9) system has emerged as a powerful tool for GE in both iPSCs and HSPCs to correct inborn errors of immunity (IEI) (128, 129). While the clinical application of gene editing in TI-IFN is still under investigation (130), SAVI may benefit from a combined approach. This could involve using INDEL-mediated gene editing (INDEL-GE) to disrupt the activity of the hyperactive mutant STING protein and homology-directed repair gene editing (HR-GE) to insert a functional, codon-optimized STING cDNA (coSTING1) at the endogenous transcriptional start site (131). Another approach might involve the use of prime and base editors, that avoid double-strand DNA breaks reducing the risk of genotoxicity (132, 133). While GE hold paradigm-shifting potential for monogenic inborn errors of immunity like SAVI, their translation into clinical practice faces profound safety, delivery, and immunological hurdles (134). Specifically, off-target genome editing and the induction of double-strand breaks (DSBs) can promote oncogenic chromosomal translocations and tissue-wide genotoxicity (135). In the context of SAVI, where patients already suffer from progressive and fragile ILD, accidental off-target cuts in surviving alveolar cells might compromise residual tissue repair mechanisms and increase respiratory failure. To mitigate these risks, advanced platforms such as Base Editors and Prime Editors have been developed to bypass DSBs and minimize stochastic insertions/deletions (indels) (136). However, these machinery constructs are molecularly massive, creating severe delivery challenges regarding viral vector packaging limits (e.g., AAV capacity). Furthermore, because SAVI is a multi-systemic disorder affecting both the vascular endothelium and deep pulmonary parenchyma (137–139). Systemic or local delivery vectors must successfully cross severely damaged, fibrotic, and poorly perfused tissue barriers. From a long-term safety perspective, permanent genomic modifications carry the risk of clonal hematopoiesis or irreversible tissue scarring—meaning GE can halt active inflammation but cannot reverse established pulmonary fibrosis (140, 141). Finally, immunogenicity represents a critical bottleneck: the hyperactive TI-INF environment and chronically primed innate immune cells of SAVI patients can react to viral capsids or editing-enzymes, triggering life-threatening cytokine release syndromes or macrophage activation syndrome (MAS) (142–145). These precision tools, if successfully translated, could finally offer a definitive cure by addressing the underlying genetic cause rather than merely managing the downstream interferon storm (146, 147). Furthermore, humanized pre-clinical models utilizing STING1-engineered HSPCs have recently established the minimum therapeutic allelic correction threshold required to completely abrogate pathogenic TI-IFN signaling (147). These quantitative benchmarks provide developers with exact target efficacy metrics, directly addressing regulatory concerns regarding sub-therapeutic editing or partial chimeric rescue in highly inflamed pediatric microenvironments.

To bypass the classic delivery bottlenecks of these macromolecular precision tools into deep tissue beds, engineering innovations have shifted toward orthogonally-optimized, endothelium-targeted, and inhalable lipid nanoparticle (LNP) platforms (148). In preclinical models, these next-generation non-viral vehicles have successfully bypassed systemic hepatic clearance, delivering *STING1*-correcting or -silencing machinery directly into alveolar macrophages and pulmonary endothelial structures, markedly reducing fibrotic deposition and cellular stress (149). To safely facilitate these interventions, a pharmacological bridging strategy utilizing JAKi, (ruxolitinib/baricitinib) has been proposed (150–152). By interrupting the autocrine/paracrine TI-IFN feedback loop upstream of JAK/STAT signaling, JAKi pre-treatment lowers the activation threshold of innate immune sensors, dampens macrophage reactivity, and protects target cells from vector-induced cytotoxicity during the genetic correction window (153, 154).

Beyond direct STING1 genetic mutations, the role of epigenetic regulation in SAVI remains an unexplored frontier. Future studies could focus on analyzing DNA methylation in the *STING1* promoter in SAVI patients to identify disease-associated methylation patterns or histone modifications in *STING1* to understand how chromatin structure could also modulate *STING1* expression (155).

#### 10.2.3 Research priorities and clinical translation

Despite recent therapeutic breakthroughs, translating these molecular discoveries into standard clinical practice requires addressing key future research priorities. First, shifting toward a genotype-guided precision medicine framework is essential, as distinct *STING1* structural variants exhibit heterogeneous pathway dependencies that may dictate tailored pharmacological responses. Second, discovering and validating biomarkers beyond the classic IFN-signature—such as comprehensive cytokine profiles, markers of ER stress, and T-cell cytotoxicity assays—would substantially improve prognostic accuracy and treatment monitoring. Third, given the extreme rarity of SAVI, establishing larger international collaborative registries and multicenter datasets is paramount to pool clinical outcomes regarding advanced therapies like anifrolumab and allo-HSCT. Finally, advancing gene therapy and editing clinical trials would require rigorous pre-clinical optimization to overcome delivery hurdles, off-target effects, long-term safety concerns (including risks of clonal hematopoiesis), ethical considerations regarding pediatric informed consent and regulatory barriers, which include three primary challenges: first, the n-of-1 nature of ultra-rare genetic variants complicates the standardization of clinical trial endpoints and statistically powered efficacy assessments; second, the requirement for ultra-stringent quality control (QC) and GMP-grade manufacturing for highly personalized genetic constructs introduces prohibitive economic costs and scaling limitations that are not sustainable under traditional commercial models; and third, current regulatory frameworks often struggle to balance the need for rapid patient access to potentially life-saving orphan therapies with the necessity of long-term longitudinal data to monitor for delayed adverse events, such as insertional mutagenesis or persistent immunogenicity. Ultimately advancing the development of curative options for affected individuals and promoting more targeted, effective treatments that address the underlying genetic cause (112, 156, 157).

Collectively, these approaches may promote more targeted and effective treatments that might address the underlying genetic cause of SAVI, ultimately improving the prognosis for affected individuals.

## 11 Conclusion

In conclusion, while the journey from bench to bedside is complex, the transition toward personalized medicine offers the most concrete hope for improving the prognosis and quality of life for individuals living with SAVI. Translating recent molecular breakthroughs into individualized clinical management requires a coordinated global effort focused on several key future research directions. Specifically, the establishment of international multicenter registries and prospective longitudinal studies will be paramount to pool clinical outcomes, standardize validated disease activity and outcome measures, and evaluate advanced interventions such as anifrolumab and allogeneic HSCT. Concurrently, rigorous validation of dynamic secondary biomarkers alongside core interferon signatures will refine treatment monitoring, while the progression toward genotype-specific clinical trials and mutation-based treatment development will enable precision therapies tailored to distinct structural STING1 variants. Ultimately, bridging these research gaps represents the decisive key to transforming SAVI from a life-threatening systemic vasculopathy into a manageable, precisely targeted condition.

## Glossary

allo-HSCT: Allogeneic hematopoietic stem cell transplantation
AMPK: AMP-activated protein kinase
ANA: Anti-nuclear antibodies
ANCA: Anti-neutrophil cytoplasmic antibodies
ASO: allele-specific antisense oligonucleotides
BLT: Bilateral lung transplantation
Cdks: Cyclin-dependent kinases
CDN: Cyclic dinucleotide
cGAMP: Cyclic GMP-AMP
cGAS: Cyclic GMP-AMP synthase
COPII: Coatomer protein complex II
CRISPR/Cas9: Clustered regularly interspaced short palindromic repeats-associated protein 9
CRP: C-reactive protein
CTD: C-terminal domain
CTT: Carboxy-terminal tail
DCs: Dendritic cells
DD: Dimerization domain
DMARD: Disease-modifying anti-rheumatic drugs
ER: Endoplasmic reticulum
ERGIC: ER-Golgi intermediate compartment
ESR: Erythrocyte sedimentation rate
GE: Gene editing
GOF: Gain-of-function
GvHD: Graft-versus-host disease
GAS: IFN-γ-activated sequences
HAQ: His-Ala-Gln (STING allele)
HSC: Hematopoietic stem cell
HSPCs: Hematopoietic stem and progenitor cells
HLA: Human leukocyte antigen
IFI16: IFN-γ-inducible protein 16
IFN: Interferon
IFNAR: Type I interferon receptor
IFNs: Interferons
ILD: Interstitial lung disease
IL-6: Interleukin-6
INDEL-GE: Insertion/deletion-mediated gene editing
iPSCs: Induced pluripotent stem cells
ISGF3: IFN-stimulated gene factor 3
ISGs: Interferon-stimulated genes
ISRE: IFN-stimulated response element
IVIG: Intravenous immune globulin
JAK: Janus Kinases
JAKi: JAK inhibitors
LNP: inhalable lipid nanoparticle
MAVS: Mitochondrial antiviral signaling protein
NF-κB: Nuclear factor kappa-light-chain-enhancer of activated B cells
NLRs: NOD-like receptors
NK: Natural killer cells
PRRs: Pattern Recognition Receptors
RLRs: RIG-I-like receptors
SAVI: STING-associated vasculopathy with onset in infancy
SCID: Severe combined immunodeficiency
siRNA: small interfering RNAs
STAT: Signal transducers and activators of transcription
STING: Stimulator of interferon genes
STIM1: Stromal interaction molecule 1
TBK1: TANK-binding kinase 1
TI-IFN: Type I interferonopathies
TLRs: Toll-like receptors
TMs: Transmembrane domains
TNFα: Tumor necrosis factor α
ULK1: Unc-51-like autophagy activating kinase 1
WES: Whole exome sequencing
