# Correction: SAVI: molecular mechanisms, clinical spectrum and precision medicine approaches beyond type-I IFN

**DOI:** 10.3389/fimmu.2026.1988994

**Published:** 2026-10-06

**Authors:** Michele Manganelli, Paola Cantalice, Jona Papri, Enrico Drago, Nicole De Lucchi, Giulia Accorsi, Chiara Fresia, Simonetta Simonetti, Gabriella Guida, Gerardo Cazzato, Mario Della Mura, Giuseppe Ingravallo, Roberta Caorsi, Stefano Volpi, Francesco La Torre, Marco Gattorno

**Affiliations:** 1 Department of Precision and Regenerative Medicine and Ionian Area, University of Bari Aldo Moro, Bari, Italy; 2 Pediatric Rheumatology Unit, Giovanni XXIII Children’s Hospital, Azienda Ospedaliero Universitaria Consorziale (AOU) Policlinico-Giovanni XXIII, University of Bari Aldo Moro, Bari, Italy; 3 Dipartimento di Neuroscienze, Riabilitazione, Oftalmologia, Genetica e Scienze Materno-Infantili, Università degli Studi di Genova, Genoa, Italy; 4 Clinical and Experimental Immunology, IRCCS Istituto Giannina Gaslini, Genoa, Italy; 5 Gene Therapy Program, Dana Farber/Boston Children’s Cancer and Blood Disorders Center, Harvard Medical School, Boston, MA, United States; 6 Clinical Pathology and Neonatal Screening, Giovanni XXIII Children’s Hospital, Azienda Ospedaliero Universitaria Consorziale (AOU) Policlinico-Giovanni XXIII, University of Bari Aldo Moro, Bari, Italy; 7 Department of Translational Biomedicine and Neuroscience, University of Bari Aldo Moro, Bari, Italy; 8 Section of Molecular Pathology, Department of Precision and Regenerative Medicine and Ionian Area, University of Bari Aldo Moro, Bari, Italy; 9 Rheumatology and Autoinflammatory Diseases Unit, IRCCS Istituto Giannina Gaslini, Genoa, Italy

**Keywords:** anifrolumab, interstitial lung disease (ILD), Janus kinase inhibitors (JAK inhibitors), STING-associated vasculopathy with onset in infancy (SAVI), type I interferonopathy, vasculopathy

An incorrect **Funding** statement was provided.

The correct funder is “Finanziato dall’Unione europea – Next Generation EU – PNRR M6C2 - Investimento 2.1 Valorizzazione e potenziamento della ricerca biomedica del SSN, codice progetto PNRR-POC-2023-12377470”.

The correct **Funding** statement reads:

“The author(s) declared that financial support was received for this work and/or its publication. This research was supported by funds from Finanziato dall’Unione europea – Next Generation EU – PNRR M6C2 - Investimento 2.1 Valorizzazione e potenziamento della ricerca biomedica del SSN, codice progetto PNRR-POC-2023-12377470.”

The original version of this article has been updated.

