# Supplementary material for: SAVI: molecular mechanisms, clinical spectrum and precision medicine approaches beyond type-I IFN
Source: Front Immunol. 2026 Aug 31;17:1928419. doi: 10.3389/fimmu.2026.1928419 (PMC13572920; doi:10.3389/fimmu.2026.1928419)
Supplement: Supplementary file 1 [file Table1.docx]

Supplementary Material

SAVI: molecular mechanisms, clinical spectrum and precision medicine approaches beyond Type-I IFN

Michele Manganelli, Paola Cantalice, Jona Papri, Enrico Drago, Nicole De Lucchi, Giulia Accorsi, Chiara Fresia, Simonetta Simonetti, Gabriella Guida, Gerardo Cazzato, Mario Della Mura, Giuseppe Ingravallo, Roberta Caorsi, Stefano Volpi, Francesco La Torre, Marco Gattorno

**Supplementary Table S1.** Review of reported SAVI patients with dominant mutation.

| **Reference** | **Case number (sex distribution), age onset** | **Lung involvement [number of patients affected]** | **Development retardation** | **Cutaneous presentations [number of patients affected]** | **Inflammatory markers** | **Immune perturbation** | **Autoantibody profile [number of patients affected]** | **Gene variant [number of patients affected]** | **Treatment** | **Prognosis** |
| --- | --- | --- | --- | --- | --- | --- | --- | --- | --- | --- |
| (12) | 6 (3 F/ 3 M), 1 day to 8 w | ILD [5], paratracheal adenopathy [6], lung fibrosis [3] | Unknown | Acral violaceous plaques [6], skin nodules [6], nail dystrophy or loss [6], nasal-septum perforation [4] | Unknown | Unknown | ANA+ [3], Antiphospholipid antibodies+ [5], c-ANCA+ [1] | de novo p.N154S [4], de novo p.V155M [1], de novo p.V147L [1] | Glucocorticoid, DMARD, Biological agent | No response or incomplete response |
| (52) | 1 (M), 1 w | ILD, pulmonary hypertension | Unknown | Ulceration of the extremities | Increased levels of CRP, ESR | Unknown | Anticardiolipin IgG+, lupus anticoagulant+ | de novo p.V155M | Corticosteroids, cyclophosphamide, azathioprine, methotrexate, rituximab and infliximab | Deceased (at 16y) |
|  | 1 (M), 65y (proband's grandpa) | Normal | yes | Unknown | Increased levels of CRP, ESR | Unknown | ANA 1:340 | p.V155M | Nil | Unknown |
| (50) | 1 (M), 29y (proband's father) | IDL | yes | Unknown | Increased levels of CRP, ESR | Unknown | ANA 1:640 | Inherited p.V155M | Nil | Deceased |
|  | 1 (M), 33y (proband's uncle) | ILD | yes | Unknown | Increased levels of CRP, ESR | Unknown | ANA 1:320 | Inherited p.V155M | Nil | Unknown |
|  | 1 (F), 4y (proband) | ILD | yes | Unknown | Increased levels of CRP, ESR | Unknown | ANA 1:200 - 1:800 | Inherited p.V155M | Nil | Unknown |
| (53) | 1 (M), 38w | ILD stable | yes | Skin necrosis, gangrene, nasal-septum perforation | Normal | Unknown | ANCA 1:200 - 1:400 | de novo p.V147M | Methylprednisolone, prednisone, clopidogrel bisulfurate, nifedipine | Pulmonary feature stable, skin lesions worse |
| (80) | 1 (M), 12y | Pulmonary fibrosis | yes | Telangiectasia and chilblains | Increased levels of CRP, ESR | CD4+ lymphopenia, NK cell decreased, hypergammaglobulinemia (IgG, IgA) | ANA+ | de novo p.V155M | Nil | Unknown |
|  | 1 (F), 20y | Pulmonary fibrosis, emphysema | Unknown | Acral telangiectasia, atrophic plaques, nail dystrophy | Unknown | CD4+ lymphopenia, NK cell decreased, hypergammaglobulinemia (IgG) | ANA+, c-ANCA+ | de novo p.V155M | Lung transplantation | Deceased |
|  | 1 (M), 5m | ILD | yes | Telangiectasia and chilblains | Increased levels of CRP, ESR | CD4+ and CD8+ lymphopenia, hypergammaglobulinemia (IgG) | Nil | de novo p.V155M | Nil | Unknown |
| (66) | 1 (M), 12y (reported by Picard et al., 2016) | Lung fibrosis | yes | Chilblains of feet and left ear | Increased levels of CRP, ESR | CD4+ lymphopenia, NK cell decreased, hypergammaglobulinemia (IgG, IgA) | ANA+ | de novo p.V155M | Hydroxychloroquine, steroids, ruxolitinib | Improved for lung symptoms but not skin lesions |
|  | 1 (F), neonate (reported by Jeremiah et al., 2014) | ILD | yes | Occasional erythema of the cheeks | Increased levels of CRP, ESR | Unknown | ANA 1:200 - 1:800 | Inherited p.V155M | Steroids, MMF, anti-CD20 monoclonal antibody, ruxolitinib | Improvement of interstitial pneumonitis |
| (70) | 1 (M), 9y | Obliterative bronchiolitis | Unknown | Perforated nasal-septum, gangrenous lesions | Increased levels of CRP, ESR | CD4+ lymphopenia, monocytes increased | Unknown | de novo p.S102P, de novo p.F279L | Tofacitinib | Only skin lesion improved |
| (105) | 1 (M), 5w | ILD | Unknown | Maculo papular, erythematous lesions | Increased levels of CRP | IgG and IgA increased | SSA+ | de novo p.V155M | Methylprednisolone and immunoglobulin | Improved |
| (158) | 1 (F), 25y | Normal | yes | Livido Acrocyanosis, palatal/nasal-septum necrosis | Normal | Unknown | ANA 1:160, ANCA- | de novo p.R284G | Nil | Unknown |
|  | 1 (F), 3m | ILD | yes | Early-onset malar rash | Increased of inflammatory markers | Unknown | Nil | de novo p.R281Q | Steroids, methotrexate, anti-TNF-α | No efficacy |
|  | 1 (M), 15y | Normal | no | Livido and dark purple marbling on the limbs, painful acral-erosions on the ear helices and digitals | Normal | Normal | Nil | de novo p.C206Y | nifedipin, pentoxyfillin, acetyl, salicylic acid | Limited efficacy for skin lesions |
| (159) | 1 (M), 3y | ILD, pulmonary hypertension | yes | Acral erythema-tous papules, livido racemosa | Increased levels of CRP, ESR, ferritin, IL-6,  IL-1β | CD4+ and CD8+ lymphopenia | Unknown | de novo p.R284S | Ruxolitinib (5mg/die and oral prednisolone (2mg/die) | Exercise capacity improved, complete loss of nasal-septum |
| (72) | 1 (M), 9y | Normal | no | No | Increased levels of IL-6 | Unknown | Unknown | de novo p.R284S | Nil | Deceased |
| (67) | 4 (unknown), 0-118y | ILD [4] | yes | Cutaneous vasculitis [4], ulcers [3] | Increased levels of CRP, ESR | Unknown | Unknown | de novo p.N154S | Baricitinib | Improved for lung function |
| (106) | 1 (M), 2m | ILD | yes | Cold-induced violaceous papules or plaques on ears | Increased levels of CRP, ESR, IL-6, TNF-α | Hypergammaglobulinemia (IgG, IgA) | RF+ | de novo p.V155M | Tafacitinib | Improved for lung function |
| (160) | 1 (F), 5y | ILD | yes | Atrophic scars, destructed nasal-septum, destructed nails | Increased levels of CRP, ESR | Unknown | ANA+ | de novo p.N154S | Methotrexate, prednisolone | Improved for lung disease and cutaneous lesions |
| (69) | 1 (F), 8m | ILD | yes | Maculopapular lesions evolving to ulcers | Increased levels of CRP, ESR | Normal | ANA+, p-ANCA+, PR3+ | de novo p.V155M | Ruxolitinib | Improved for lung disease and cutaneous lesions |
|  | 1 (F), 3m | ILD | yes | Malar rash | Increased levels of CRP, ESR | Normal | c-ANCA+, anti-cardiolipin+, B2GlG+ | de novo p.R281Q | Ruxolitinib | Skin lesions and ILD worsened |
|  | 1 (F), 3m | ILD | yes | Erythematous vesicular rash | Increased levels of CRP, ESR | Normal | c-ANCA+, PL+, anti-cardiolupin+, PT+ | de novo p.N154S | Ruxolitinib | Transient improvement for lung disease and skin lesions, and then lapsed of ILD |
| (71) | 1 (M), 6m | ILD | no | Purplish red non-indurating plaques and pustular lesions | Increased levels of CRP, ESR | Normal | Nil | de novo p.N154S | Ruxolitinib switch to baricitinib | Greatly improved for lung and cutaneous lesions |
| (74) | 1 (F), 4m | ILD | yes | No | Increased levels of ESR | Hypergammaglobulinemia IgG and decreased NK cells, decreased CD3+ lymphocytes and CD3+ CD8+ lymphocytes | ANCA+ (25m after the onset), ANA 1:80 | de novo p.V155M | Corticosteroids, IVIG | Improved for lung and cutaneous lesions |
|  | 1 (F), 3m | ILD | yes | Angiotelectasis, chilblain lesions | Normal | Hypergammaglobulinemia IgG and decreased NK cells, decreased CD3+ lymphocytes and CD3+ CD8+ lymphocytes | Unknown | de novo p.V155M | Corticosteroids, IVIG, tofacitinib | Deceased |
|  | 1 (M), 54m | ILD | no | Angiotelectasis, erythematous, purpuric or scaling rashes | Increased levels of CRP, ESR | Hypergammaglobulinemia IgG and decreased NK cells | RF+, CCP+ | de novo p.V155M | Corticosteroids, IVIG, CTX, tofacitinib | Respiratory symptoms improved but rashes worsened |
| (75) | 1 (M), birth | Normal | Unknown | Livido reticularis, skin vasculitis, nasal-septal perforation, facial erythema, UV sensitivity, alopecia, skin infections | Unknown | Decreased CD19+ B cells and NK cells, increased IgE | Negative | Inherited p.G207E | Prednisolone combined with azathioprine or methotrexate, cyclosporine, baricitinib | Improved for alopecia and over-well being |
|  | 1 (F), birth | Recurrent pneumonia | Unknown | Livido reticularis, skin vasculitis, nasal-septal perforation, facial erythema, UV sensitivity, alopecia, skin infections, autoimmune thyroiditis | Unknown | Increased IgE | Negative | Inherited p.G207E | Antibiotics, prednisolone and cyclosporine | Improved for skin infection |
|  | 1 (F), birth | Normal | Unknown | Livido reticularis, facial erythema, UV sensitivity, alopecia, skin infections, hypothyroid | Unknown | Increased IgE | Negative | Inherited p.G207E | Unknown | Unknown |
|  | 1 (F), birth | Normal | Unknown | Livido reticularis, nasal-septal perforation, facial erythema, UV sensitivity, alopecia, autoimmune thyroiditis | Unknown | Normal | Negative | Inherited p.G207E | Unknown | Unknown |
|  | 1 (F), birth | Normal | Unknown | Livido reticularis, facial erythema, UV sensitivity, skin infections, periodontitis | Unknown | Increased IgE | Negative | Inherited p.G207E | Unknown | Unknown |
|  | 1 (F), birth | Normal | Unknown | Livido reticularis, UV sensitivity, alopecia, autoimmune thyroiditis, periodontitis | Unknown | Increased IgE | Negative | Inherited p.G207E | Radioiodine treatment, thyroid hormone substitution, calcium and vitamin D-replacement therapy | Improved for alopecia |
| (161) | 1 (M), 2m | ILD | yes | Rash | Increased levels of CRP, ESR | Increased IgE, hypergammaglobulinemia IgA | Negative | de novo p.V155M | Unknown | Unknown |
|  | 1 (M), 1m | ILD | yes | Telangiectatic skin lesions | Increased levels of CRP, ESR | Increased IgE, hypergammaglobulinemia IgA, decreased IgG and IgM | CCP+ | de novo p.N154S | Unknown | Unknown |
| (81) | 1 (F), neonate | ILD | yes | No | Increased levels of CRP | Hypergammaglobulinemia (IgG, IgA and IgM) | Anti-smooth muscle+ | Inherited p.V155M | Methylprednisolone, IVIG, baricitinib | Improved for lung disease |
|  | 1 (F), 22m | ILD | Unknown | Rash | Normal | Hypergammaglobulinemia (IgG, IgA and IgM) | ANA 1:320, RF+ | Inherited p.V155M | Oral prednisolone, rituximab, baricitinib | Improved for lung disease |
|  | 1 (F), 16y | ILD | Unknown | Unknown | Normal | Hypergammaglobulinemia IgA | ANA 1:80/negative, Anti-dsDNA+, RF+ | de novo p.V155M | Oral and intravenus steroids | Deceased |
| (162) | 1 (M), 4w | ILD | yes | Blisters, ulcers, digital ischemia, nephroticrange proteinuria | Increased levels of inflammatory markers | Unknown | Unknown | de novo p.V147L | Ruxolitinib, methylprednisolone | Transient improvement for skin lesions and lung disease |
| (57) | 1 (M), 4w, proband A | ILD | yes | Macupapular violaceous rash with a livedoid appearance | Unknown | Unknown | Unknown | p.R281W | Steroids, tofacitinib | Deceased |
|  | 1 (M), 18m (proband A's brother) | Chronic cough | yes | Unknown | Unknown | Unknown | Unknown | Unknown | Unknown | Deceased |
|  | 1 (M), 3m, proband B | ILD | Unknown | Erythematous rash | Unknown | Unknown | Unknown | p.R281W | Ruxolitinib | Unknown |
|  | 1 (M), 6m (proband B's brother) | Unknown | Unknown | Unknown | Unknown | Unknown | Unknown | p.R281W | Steroids, baricitinib | Unknown |
|  | 1 (F), 2m | ILD, recurrent lung infections and pulmonary hypertension | Unknown | Erythematous rash and polyarthritis | Unknown | Unknown | Unknown | p.R281W | Baricitinib | Clinical improvement, still oxygen dependence |
|  | 1 (M), 8m | Unknown | yes | Intermitted vasculitic rash and polyarthritis | Unknown | Unknown | Unknown | p.R281W | Unknown | Unknown |
| (96) | 1 (M), 37y | ILD | no | Clubbing fingers, nails, dystrophy of toes | Increased levels of inflammatory markers | Hypergammaglobulinemia IgA, increased CD8+ cells | Multiple positive antibodies | de novo p.R281W | Ruxolitinib | Deceased |
|  | 1 (M), 13y | ILD | yes | Malar rash | Elevated | Unknown | ANA 1:1280 | Inherited p.R281W | Ruxolitinib | No improvement in polyarthritis |
|  | 1 (M), 6y | ILD | no | Angiotelectasis | Elevated | Unknown | ANA 1:320 | Inherited p.R281W | Nil | Nil |

F, female; M, male; y, years; w, weeks; ILD, interstitial lung disease; ANA, anti-nuclear antibodies; ANCA, Anti-neutrophil cytoplasmic antibodies; DMARD, Disease modifying anti-rheumatic drugs; CRP, C-reactive protein; ESR, Erytrocyte sedimentation rate; SSA, anti- Sjögren’s-syndrome-related antigen A antibodies; TNF-α, Tumor necrosis factor-α; IL-6, interleukin 6; IL-1β, Interleukin 1β; IgG, Immunoglobulin G; IgA, Immunoglobulin A; IgE, Immunoglobulin E; IgM, Immunoglobulin M; RF, Rheumatoid factor; PR3, anti-proteinase 3 antibodies; B2Gl2, anti-β-2 glycoprotein; PL, anto-phospholipid antibodies; PT, anti-prothrombin antibodies; CCP, anti-cyclic citrullinated peptide antibodies; IVIG, Intravenous immune globulin.

**Supplementary Table S2.** Review of reported SAVI patients with recessive mutation.

| **Reference** | **Case number (sex distribution), age onset** | **Lung involvement [number of patients affected]** | **Development retardation** | **Cutaneous presentations [number of patients affected]** | **Inflammatory markers** | **Immune perturbation** | **Autoantibody profile [number of patients affected]** | **Gene variant [number of patients affected]** | **Treatment** | **Prognosis** |
| --- | --- | --- | --- | --- | --- | --- | --- | --- | --- | --- |
| (59) | 1 (M), 5m | ILD | Unknown | none | Normal | Increased IgE | ANA +, ANCA+ | Inherited p.R281W | initially hydroxychloroquine (10mg/d/kg) orally, steroid pulse therapy, then baricitinib, up to 0.5 mg/kg p.o. daily | progression of pulmonary fibrosis |
|  | 1 (M), 12days | ILD, lung fibrosis, bronchiectasis | Unknown | facial telangiectatic lesions/rash | Normal | Increased IgG2 | anti-DNA+, anti-ENA+, anti-ENA ss-A/ro | Inherited p.R281W | steroid-pulse therapy, then ruxolitinib up to 1 mg/kg p.o. daily | unknown |
|  | 1 (M), 12m | ILD | Unknown | suspected vasculitis lesion during infancy | Unknown | Increased IgE | ANA +, ANCA+ | Inherited p.R281W | steroid pulse therapy | N/A |
|  | 1 (F), unknown | ILD | Unknown | none | Unknown | Increased IgM | ANA + | Inherited p.R281W | none | N/A |
| (57) | 1 (M), 4w | ILD | Unknown | a livedoid appearance rash | Increased levels of CRP | Nil | ANA + | Inherited p.R281W | steroid therapy, short-term treatment with tofacitinib | †patient died due to respiratory failure at 5 months of age |
|  | 1 (M), unknown | Chronic cough, failure to thrive | Unknown | unknown | Unknown | Increased IgM | unknown | Inherited p.R281W | none | †patient died due to failure to thrive at 18 months of age |
|  | 1 (M), 3m | ILD | Unknown | erythematous rash | Normal | Nil | ANA+, anti-PR3+ | Inherited p.R281W | ruxolitinib | N/A |
|  | 1 (M), 6m | ILD | Unknown | facial rash | Normal | Increased IgG | ANA+,  anti-MPO+ | Inherited p.R281W | steroid treatment, baricitinib | N/A |
|  | 1 (F), 2m | Suspicious for ILD, pulmonary hypertension, recurrent lung infections, | Unknown | plantar erythematous rash | Normal | Increased IgG | ANA+, ANCA+ | Inherited p.R281W | baricitinib at the age of 7 years | N/A |
|  | 1 (M), 8m | Suspicious for ILD, cystic lung lesions, fibrosis, failure to thrive, | Unknown | intermittent vasculitic rashes | Normal | Increased IgM | ANA+, ANCA+ | Inherited p.R281W | baricitinib | N/A |
| (58) | 1 (M), unknown | Suspicious for ILD, pulmonary hypertension | Unknown | violet-red skin rash | Normal | Increased IgG | ANA + | Inherited p.R281W | systemic steroid treatment, then ruxolitinib | reduced skin lesion and pulmonary phenotype |
|  | 1 (F),  1-3y | Suspicious for ILD, diffuse bilateral opacities, pulmonary hypertension | Unknown | malar, facial rash | Normal | Increased IgG | ANA+, ANCA+, anti-DNA+ | Inherited p.R281W | steroid treatment, methotrexate, hydroxychloroquine, then ruxolitinib | reduction in the size of the area of hair loss with hair regrowth and a decrease in the hypopigmenta |

F, female; M, male; y, years; w, weeks; ILD, interstitial lung disease; ANA, anti-nuclear antibodies; ANCA, Anti-neutrophil cytoplasmic antibodies; CRP, C-reactive protein; ESR, Erytrocyte sedimentation rate; TNF-α, Tumor necrosis factor-α; IL-6, interleukin 6; IL-1β, Interleukin 1β; IgG, Immunoglobulin G; IgA, Immunoglobulin A; IgE, Immunoglobulin E; IgM, Immunoglobulin M; RF, Rheumatoid factor; PR3, anti-proteinase 3 antibodies; B2Gl2, anti-β-2 glycoprotein; PL, anto-phospholipid antibodies; PT, anti-prothrombin antibodies; CCP, anti-cyclic citrullinated peptide antibodies; IVIG, Intravenous immune globulin.

**Supplementary Table S3.** STING1 allelic variants and their prevalence in different ethnicities.

| **STING1 Variant** | **Amino Acid Changes** | **cGAMP Responsiveness (TI-IFNs)** | **cGAMP Responsiveness (T Cell Death)** | **Prevalence by Ethnicity** | **References** |
| --- | --- | --- | --- | --- | --- |
| R232 (Major Allele) | None | Responsive | Responsive | Most prevalent in Europeans;  <50% in Americans | (163–165) |
| HAQ (minor allele) | R71H, G230A, R293Q | Reduced / Hyporesponsive (Controversial in early in vitro assays, resolved by human KI models) | Defective / Resistant to cGAMP-induced T cell death | Dominant allele in East Asians;  absent in African populations;  occurs in other populations | (163–167) |
| AQ | G230A, R293Q | Variable | Defective / Resistant to T cell death | African populations | (60,163–167) |
| Q293 | R293Q | Non-responsive | Resistant to T cell death | African populations | (163–167) |
| CDN-sensing-defective variants (R232H, HAQ, AQ, Q293 combined) | Various | Variable | Defective | ~10% in Europeans;  ~30% in East Asians | (167) |

**Supplementary Table S4.** Quantitative synthesis of reported SAVI cases: mutation spectrum, clinical manifestations, and treatment outcomes.

| **Clinical Domain** | **Literature findings** | **Dominant vs. Recessive Distribution** | **Key *STING1* Variants Involved** | **References** |
| --- | --- | --- | --- | --- |
| **Demographics and Genetics** | Total cohorts: ~40–50 individual pediatric and adult cases with deep clinical mapping.    High prevalence of de novo mutations in early-onset severe cases; familial transmission documented in milder or adult-onset phenotypes. | Dominant: ~80–85% of cases.    Recessive: ~15–20% of cases (primarily linked to homozygous/compound heterozygous states). | V155M (most frequent), N154S, R281W, V147L, G207E, R284S/G, S102P, F279L, C206Y. | Aggregated data from literature review (detailed case-by-case mapping in Supp. Tables S1 and S2 |
| **Pulmonary Involvement (ILD)** | Prevalence: >85–90% of symptomatic published cases.    Spans progressive interstitial lung disease (ILD), pulmonary fibrosis, pulmonary hypertension, and recurrent respiratory infections leading to respiratory failure. | Present across both dominant and recessive inheritance patterns with variable age of onset. largely absent in modified asymptomatic carriers. | V155M, N154S, R281W, V147L. | Aggregated data from literature review (detailed case-by-case mapping in Supp. Tables S1, S2 and S4 |
| **Cutaneous Manifestations** | Prevalence: ~80% of symptomatic cases.    Characterized by acral violaceous plaques, severe chilblains, skin ulcers, necrosis, digital gangrene, and characteristic nasal-septum perforation. | Frequently prominent in early-onset dominant forms (V155M, N154S). | V155M, N154S, V147L, R281S, S102P. | Aggregated data from literature review (detailed case-by-case mapping in Supp. Tables S1 and S2 |
| **Immunological Profile and Autoimmunity** | ANA Positivity: Observed in ~50–60% of cases.    ANCA Positivity: Reported in ~30% of patients, frequently mimicking systemic vasculitis.    Cellular Defects: CD4+/CD8+ lymphopenia, NK cell reduction, T-reg depletion, and hypergammaglobulinemia (IgG, IgA, IgE). | Consistent across the major pathogenic gain-of-function variants; mitigated in presence of common modulatory alleles. | All major genotypes. | Aggregated data from literature review (detailed case-by-case mapping in Supp. Tables S1 and S2 |
| **Therapeutic Response** | Agents used: Ruxolitinib, baricitinib, and tofacitinib.    Outcomes: Variable efficacy; frequent partial-to-good improvement in cutaneous lesions and stabilization of lung function, though progressive pulmonary fibrosis can persist in refractory subsets. | Efficacy is independent of inheritance mode but heavily reliant on early intervention before irreversible fibrosis sets in. | V155M, N154S, R281W, S102P. | Aggregated data from literature review (detailed case-by-case mapping in Supp. Tables S1 annd S2 |
| **End-Stage Management and Prognosis** | High morbidity and mortality due to end-stage pulmonary fibrosis.    Advanced interventions: Bilateral lung transplantation (BLT) and allogeneic hematopoietic stem cell transplantation (allo-HSCT) to correct the hematopoietic compartment. Post-transplant survival challenged by viral reactivations (e.g., BKV) and infections. | Documented primarily in severe V155M and R281Q cases progressing despite JAKi therapy. | V155M, R281Q. | Aggregated data from literature review (detailed case-by-case mapping in Supp. Tables S1, S2 and S4 |

**Supplementary Table S5.** Genetic, clinical phenotypes, molecular pathophysiology, and therapeutic paradigms of monogenic type I interferonopathies (TI-IFNs).

| **Feature** | **SAVI (STING1;**  **OMIM: #615934)** | **COPA Syndrome (COPA;**  **OMIM: # 616414)** | **Aicardi-Goutières Syndrome (AGS; OMIM: #225750)** | **CANDLE Syndrome (OMIM: #256040)** | **USP18 Deficiency**  **(OMIM: #617397)** | **STAT2 Gain-of-Function**  **(OMIM: #618886)** | **Referenses** |
| --- | --- | --- | --- | --- | --- | --- | --- |
| **Gene(s)** | *STING1* (GOF) | *COPA* | *TREX1, RNASEH2A/B/C, SAMHD1, ADAR1, IFIH1* (LOF or GOF for *IFIH1*) | *PSMB8, PSMB4, PSMA3* (LOF) | *USP18* (LOF) | *STAT2* (GOF) | (5,12,168,169) |
| **Inheritance** | AD (rare AR) | AD | AR (some AD) | AR | AR | AR | (5,12,168,169) |
| **Age of Onset** | Infancy (first weeks/months) | Infancy to early childhood (can range from early childhood to adulthood, often presenting in pediatric age) | Infancy/early childhood | Early childhood (often neonatal or early infancy) | Infancy (neonatal period) | Infancy (neonatal or early infancy) | (5,12,168,169) |
| **Primary Organ / Cutaneous Manifestations** | Skin (ulcers/necrosis, vasculopathy, livedo reticularis, cold-induced exacerbation), Lungs (ILD/fibrosis), Joints, Failure to thrive, anemia, Raynaud's | Lungs (ILD/alveolar hemorrhage), Joints (arthritis), Kidneys (immune complex glomerulonephritis), mild skin involvement (occasional livedo or purpura) | Brain (encephalopathy, calcifications), Skin (chilblains, acrocyanosis, panniculitis), Spasticity, dystonia, glaucoma | Skin (annular plaques, recurrent panniculitis, violaceous plaques), Lipodystrophy, Joint contractures, Fevers, Arthralgia | Febrile illness, skin rash, severe systemic autoinflammation, recurrent infections, multiorgan failure in severe neonatal forms (pseudo-TORCH) | Skin rash, recurrent fever, autoinflammation, recurrent viral infections | (5,12,168–170) |
| **Prominent Lung Involvement (ILD)** | Present and Hallmark (progressive fibrosis) | Present (resembling SAVI phenotype, interstitial lung disease, alveolar hemorrhage) | Absent / Not a primary feature (Rare) | Absent / Minimal pulmonary impact (Rare) | Not typical (can occur rarely in severe systemic inflammatory states) | Not typical | (5,12,168,169,171) |
| **Core Neurological Involvement** | Absent (rare basal ganglia changes / secondary) | Absent / Rare | Present and Hallmark (severe developmental delay, intracranial calcifications, leukoencephalopathy) | Absent / Variable mild delay | Not typical (though severe systemic inflammation can cause secondary encephalopathy) | Not typical | (5,12,168,169,172) |
| **Pathophysiology** | Constitutive STING activation 🡪IFN-dependent/independent pathways (ER stress, necroptosis, pyroptosis) | Mutant COPA protein retention in the ER 🡪aberrant STING/IRF3 pathway activation and ER stress 🡪 type I IFN induction | Accumulation of endogenous nucleic acids 🡪 cGAS/RLR activation 🡪 type I IFN overproduction | Proteasome dysfunction 🡪 accumulation of ubiquitinated proteins 🡪 ER stress 🡪 type I IFN induction | Loss of USP18 🡪 impaired negative feedback on IFNAR signaling 🡪 excessive type I IFN response | Impaired USP18 recruitment / defective interaction with STAT2 🡪 defective negative regulation of type I IFN signaling | (5,12,168,169,173,174) |
| **Type I IFN Signature in Blood** | Elevated / High | Elevated / High | Elevated / High | Elevated / High | High | High (type I IFN) | (13,40,175) |
| **Indications for Lung Transplantation** | Considered in end-stage refractory ILD / fibrotic progression | Considered in end-stage refractory fibrotic ILD / severe respiratory failure | Not Indicated (pulmonary sparing) | Not Indicated (pulmonary sparing) | Not typically indicated (pulmonary sparing, managed through immunomodulation) | Not typically indicated (pulmonary sparing) | (39,176) |
| **Primary / Typical Therapeutic Strategy** | JAKi (ruxolitinib, baricitinib), anifrolumab, supportive care, BLT or allo-HSCT | JAKi (baricitinib, ruxolitinib), conventional immunosuppression, corticosteroids, supportive care | JAKi (baricitinib), supportive care, reverse transcriptase inhibitors (investigational) | JAKi (baricitinib), systemic corticosteroids, targeted biologics | JAKi (baricitinib/ruxolitinib), supportive care, consideration of hematopoietic stem cell transplantation (HSCT) in severe cases | JAKi (baricitinib/ruxolitinib), supportive care | (68,176–179) |

AD: Autosomal Dominant, AR: Autosomal Recessive, GOF: Gain-of-Function, LOF: Loss-of-Function, OMIM: Online Mendelian Inheritance in Man, ILD: Interstitial Lung Disease, ER: Endoplasmic Reticulum, IFN: Interferon, BLT: Bilateral Lung Transplantation, allo-HSCT: Allogeneic Hematopoietic Stem Cell Transplantation, JAKi: Janus Kinase Inhibitors

**Supplementary Table S6.** Clinical Characteristics and Post-Transplant Outcomes in SAVI Patients.

| **Source (year)** | **Patient** | **Sex** | **Age**  **at LT** | **STING1 variant** | **LT** | **FEV1**  **pre-LT** | **Diagnosis** | **JAKi** | **Timing** | **Observations** | **Outcome** | **Survival after LT** | **Status at last**  **follow-up** | **Infectious** | **Thromboembolic** | **Airway / Pulmonary** | **Renal** | **Rejection** | **Reference** |
| --- | --- | --- | --- | --- | --- | --- | --- | --- | --- | --- | --- | --- | --- | --- | --- | --- | --- | --- | --- |
| **Picard (2016)** | P2 | F | 34 yo | V155M | BLT | Mixed, obstructive | After LT | None | — | No targeted therapy available; diagnosis made post-mortem | D | ~3m | Primary graft dysfunction, cholestatic hepatitis, hemophagocytic syndrome, TMA, MOF | Fungal  (fatal) | NR | PGD | Renal insufficiency | NR | (80) |
| **Clarke (2020)** | P3 | F | 30 yo | V155M | SLT | Restrictive, TF 32% | After LT | None | — | Diagnosis only confirmed post-mortem via stored tissue | D | 8y | MOF + fungal infection of transplanted lung (presented with acute respiratory failure and lower-lobe opacification) | Fungal  (fatal) | NR | ND | NR | NR | (81) |
| **Kim (2022)** | P1 | F | 17 yo | V155M | BLT | NR | After LT | Baricitinib | Post-LT only | Started after SAVI diagnosis was made post-LT; discontinued after PTLD (GI tract) developed at 4 months | A | ~4–5m | GI-PTLD developed 4 months after starting baricitinib; baricitinib discontinued and PTLD-specific treatment initiated | PTLD  (GI) | NR | Recurrent infiltrates (disease recurrence) | NR | NR | (77) |
| **Berrada (2023)** | P1 | M | 17 yo | V155M | BLT | 24.6% (0.87 L) | Before LT | Ruxolitinib | Pre- and post-LT | Transient improvement pre-LT; IFN score persistently elevated post-LT without normalization | A | 2.6y | Chilblains; FEV1 2.65 L (−1.77 Z-score, just below LLN); chronic BKV infection; renal function normalized | P. aeruginosa, A. fumigatus (recurrent); BKV | Renal vein thrombosis (M9) | Bilateral bronchial stenosis; endobronchial stenting | BKV nephropathy; CKD | None (transbronchial bx negative) | (82) |
| **Berrada (2023)** | P2 | F | 13.9 yo | V155M | BLT | 33%  (0.87 L) | Before LT | Ruxolitinib | Pre- and post-LT | Partial response pre-LT; severe VZV infection and osteonecrosis post-LT; died of bronchial wall rupture | D | 4y | Fatal massive hemoptysis from left bronchial wall rupture after coughing effort | VZV (acyclovir-resistant); BKV; Staph, H. influenzae, Citrobacter | DVT (PICC); osteonecrosis (femur/tibia) | Bronchial granulation; stent; fatal bronchial rupture at 4 y | BKVAN; hemodialysis | ACR ×2 (A3 and A2) | (82) |
| **Berrada (2023)** | P3 | M | 14.3 yo | V155M | BLT* | 16% (0.49 L) [2nd LT] | Before LT | Ruxolitinib | Pre- and post-LT | Introduced at age 12; continued through both LTs; died 33 days after 2nd LT from MOF | D | 33d  (2nd LT) | Postoperative bleeding, ARDS, VA-ECMO, humoral rejection, AKI, MOF | A. fumigatus; Candida; ESBL Enterobacter | PE + subclavian/femoral thrombosis (M17) | Diaphragmatic paresis; tracheostomy; CLAD (led to 2nd LT) | NS | Humoral rejection; rituximab ×2 | (82) |
| **Lv (2026)** | P1 | M | 28 yo | R281Q | BLT | Unable to perform | Before LT | None | — | Decision not to start JAKi pre-LT due to rapid deterioration and variable evidence; no JAKi post-LT at 5-month follow-up | A | 5m | No respiratory symptoms; mMRC 0; PaO₂ 80.4 mmHg; FEV1 52.5% predicted; regular follow-up ongoing | K. pneumoniae; P. aeruginosa; CMV | ND | ND | Normal | AMR at W3 (resolved) | (79) |

Abbreviations: yo: years old; M: male; F: female; A:alive; D: deceased; d: days; m: months; y: years; LT: lung transplantation; BLT: bilateral lung transplantation; SLT: single lung transplantation, BLT*: lung re-transplantation; JAKi: Janus kinase inhibitor; NR: not reported; NS: not specified; ND: not documented.

**Supplementary Table S7.** Summary of clinical and immunological phenotypes in SAVI murine models.

| **Pathological Feature** | **p.V154M** | **p.N153S** | **hSTING** |
| --- | --- | --- | --- |
| **Reduced Survival** | Present, more severe | Present, less severe | Present |
| **Interstitial Lung Disease (ILD)** | Inflammation and fibrosis, hallmark feature | Inflammation | Absent, severe lung disease does not develop to the same extent (limited pulmonary involvement) |
| **Immunodeficiency / SCID-like Phenotype** | Present, exceeds the severity of immune defects reported in humans; T-cell lymphopenia depends on T-cell-intrinsic expression | Present, pathology intrinsic to the hematopoietic compartment | Present (hematopoietic expression) |
| **Cells Involved** | Hematopoietic and non-hematopoietic components (stromal cells play a crucial role in initiating pulmonary inflammation; endothelial-restricted expression is insufficient) | Hematopoietic component | Hematopoietic component |
| **Interferon Pathway** | Independent | Independent | Dependent |
| **Bone Marrow / Radiation Chimera Transfer** | Bone marrow from mutant mice was able to transfer disease to wild-type hosts (WT→VM chimeras show stromal and hematopoietic interplay) | Bone marrow from mutant mice was *not* able to transfer disease to wild-type hosts | Induces systemic inflammation and vascular alterations upon expression in hematopoietic cells |

**Supplementary Table S8.** Therapeutic landscape and target mechanisms within non-genetic molecular therapies in SAVI.

| **Therapeutic Class** | **Primary Mechanism of Action** | **Molecular Target / Site** | **Key Clinical Applications** | **Developmental Status and Efficacy against V155M (SAVI)** | **Systemic Tolerability / Toxicity Profile** | **References** |
| --- | --- | --- | --- | --- | --- | --- |
| **Direct STING Inhibitors** | Block cGAMP binding or lock STING in inactive conformation.    Prevent oligomerization/trafficking to the Golgi.    Promote degradation of mutant platforms. | Cytosolic ligand-binding domain (LBD) or Transmembrane Domain (TMD). | SAVI (interferonopathies)    Systemic Lupus Erythematosus (SLE) | Phase 1/2 for orthosteric inhibitors (partial efficacy on V155M).    High efficacy for TMD allosteric wedges (e.g., Y-320 class) that mechanically arrest the pathway. | Risk of impaired host defense against DNA viruses.    Concerns regarding target-mediated systemic immunosuppression. | (108,111,113) |
| **cGAS Inhibitors** | Target the catalytic pocket to block ATP/GTP to cGAMP conversion.    Prevent formation of the cGAS-dsDNA dimer. | Orthosteric catalytic pocket of cGAS | Systemic Lupus Erythematosus (SLE)  Aicardi-Goutières Syndrome (AGS)    Rheumatoid arthritis | Phase 2 (e.g., VENT-03, Clinical Trial ID: NCT07260877) and Phase 1b (e.g., IMSB301, Clinical Trial ID: ISRCTN90049550).    No efficacy in SAVI (bypassed by constitutive V155M activation). | Generally high preclinical safety margin.    Minimal predicted off-target cytotoxicity, though altering baseline cytosolic DNA sensing requires long-term vigilance. | (112) |
| **TBK1 Inhibitors** | Target the ATP-binding pocket of kinase TBK1.    Prevent IRF3 phosphorylation and nuclear translocation. | Active kinase domain of TBK1 | Advanced solid tumors and immunotherapy resistance    Autoimmune neuroinflammation | Phase 1/2 in oncology.    Active preclinical models for cross-repurposing in autoimmune disorders. | Significant due to pleiotropic functions of TBK1.    Potential risks include hepatotoxicity, weight loss, and secondary effects on bone remodeling. | (114) |
| **RNA-Based Therapeutics** | siRNA: RISC-mediated cleavage of target mRNAs.    **ASOs:** Induce RNase H degradation or alter pre-mRNA splicing. | *STING1* transcript | Monogenic interferonopathies    Rare neuro-inflammatory mutations | Preclinical optimization (customized allele-specific constructs for SAVI). | Transient and dose-dependent profile.    Potential toxicity tied to delivery vehicles (e.g., LNP-induced acute cytokine release or hepatic clearance strain). | (117) |

ASOs: Antisense oligonucleotides; ATP: Adenosine triphosphate; cGAS: Cyclic GMP-AMP synthase; cGAMP: Cyclic GMP-AMP; dsDNA: Double-stranded DNA; GTP: Guanosine triphosphate; IRF3: Interferon regulatory factor 3; LBD: Ligand-binding domain; LNP: Lipid nanoparticle; RISC: RNA-induced silencing complex; SAVI: STING-associated vasculopathy with onset in infancy; siRNA: Small interfering RNA; SLE: Systemic lupus erythematosus; STING: Stimulator of interferon genes; TBK1: TANK-binding kinase 1; TMD: Transmembrane domain.

**Supplementary Table S9.** Advanced molecular platforms and future perspectives in SAVI.

| **Focus Area** | **Core Technological or Biological Approach** | **Specific Mechanisms / Strategies** | **Long-term Data Availability (5-10 yrs)** | **Key Clinical Implications and Challenges** | **References** |
| --- | --- | --- | --- | --- | --- |
| **Advanced Preclinical Models** | Patient-derived iPSCs and humanized models | Differentiation into innate immune cells and lung organoids.    Identification of radioresistant non-immune cell compartments (pulmonary endothelial and stromal cells) as drivers of fatal Interstitial Lung Disease (ILD). | N/A (Model validation phase; historical longitudinal human data derived primarily from patient registries rather than pre-clinical iPSC platforms). | Overcomes knowledge gaps in SAVI pathogenesis.    Establishes that therapy requires deep tissue penetration beyond hematopoietic reconstitution alone. | (97,119-121) |
| **Genome Editing (GE) Technologies** | CRISPR/Cas9 systems, Base Editors, and Prime Editors | INDEL-GE: Disrupts hyperactive mutant STING activity.    HR-GE: Inserts functional, codon-optimized *STING1* cDNA (coSTING1).    Base/Prime Editors: Avoid double-strand breaks (DSBs) to reduce genotoxicity. | Very Limited / Absent (In-human durable follow-up are restricted to early-phase trials for other monogenic targets; long-term tracking for somatic editing in lung/vascular tissues is ongoing). | Offers potential curative correction.    Challenges: Off-target cuts, oncogenic translocations, viral vector packaging limits, and immunogenicity (cytokine release or MAS). | (131,132,134,136–138) |
| **Targeted Delivery and Bridging Platforms** | Orthogonally-optimized, endothelium-targeted, and inhalable LNPs | Non-viral lipid nanoparticles (LNPs) deliver correction/silencing machinery to alveolar macrophages and pulmonary endothelium.    Pharmacological bridging: JAK inhibitors (ruxolitinib/baricitinib) dampen innate immune reactivity. | Limited / Emerging (While LNP delivery is clinically validated via vaccines, chronic pulmonary administration and targeted systemic endothelial release lack extended multi-year safety data). | Bypasses systemic hepatic clearance and fibrotic lung barriers.    JAKi pre-treatment lowers activation thresholds and protects cells from vector-induced toxicity. | (148–151) |
| **Epigenetic Regulation and Biomarkers** | Chromatin and expression profiling | Analysis of DNA methylation patterns and histone modifications in the *STING1* promoter.    Expansion of biomarkers beyond the classic IFN-signature (cytokine profiles, ER stress markers, T-cell cytotoxicity). | Ongoing Validation (Long-term biomarker stability cohorts are currently tracking longitudinal shifts during targeted biological therapies). | Explores how chromatin structure modulates *STING1* expression.    Improves prognostic accuracy and treatment monitoring. | (154) |
| **Clinical Translation and Regulatory Challenges** | Multi-stakeholder clinical and trial optimization | Genotype-guided precision frameworks.    International collaborative registries for ultra-rare variants.    Overcoming n-of-1 trial design limits, GMP economic costs, and long-term safety monitoring. | Established Historical Context (Decade-long tracking exists for traditional immunosuppression and HSCT in interferonopathies, but custom gene therapies lack mature 10-year cohorts). | Addresses regulatory and manufacturing hurdles.    Aims to bridge the gap between bench discoveries and curative, genotype-driven clinical applications. | (155–157) |

CRISPR: Clustered regularly interspaced short palindromic repeats; GMP: Good manufacturing practice; HR-GE: Homology-directed repair gene editing; INDEL-GE: Insertion/deletion gene editing; iPSCs: Induced pluripotent stem cells; LNPs: Lipid nanoparticles; MAS: Macrophage activation syndrome; N/A: Not applicable.
